# Stem cell engineering for the generation of allogeneic CAR-directed natural killer T cells targeting endometrial carcinoma

**DOI:** 10.1186/s40164-026-00746-8

**Published:** 2026-01-29

**Authors:** Yan-Ruide Li, Gabriella A. DiBernardo, Yuning Chen, Xinyuan Shen, Ryan Hon, Lauryn E. Ruegg, Jie Huang, Adam Neal, Neda A. Moatamed, Sanaz Memarzadeh, Lili Yang

**Affiliations:** 1 Department of Microbiology, Immunology & Molecular Genetics, University of California, Los Angeles, Los Angeles, CA 90095 USA; 2 Department of Bioengineering, University of California, Los Angeles, Los Angeles, CA 90095 USA; 3 Department of Obstetrics and Gynecology, David Geffen School of Medicine, University of California, Los Angeles, Los Angeles, CA 90095 USA; 4 Eli and Edythe Broad Center of Regenerative Medicine and Stem Cell Research, University of California, Los Angeles, Los Angeles, CA 90095 USA; 5 Department of Pathology and Laboratory Medicine, David Geffen School of Medicine, University of California, Los Angeles, Los Angeles, CA 90095 USA; 6 Jonsson Comprehensive Cancer Center, David Geffen School of Medicine, University of California, Los Angeles, Los Angeles, CA 90095 USA; 7 Molecular Biology Institute, University of California, Los Angeles, Los Angeles, CA 90095 USA; 8 Department of Molecular and Medical Pharmacology, David Geffen School of Medicine, University of California, Los Angeles, Los Angeles, CA 90095 USA; 9 The VA Greater Los Angeles Healthcare System, Los Angeles, CA 90073 USA; 10 Parker Institute for Cancer Immunotherapy, University of California, Los Angeles, Los Angeles, CA 90095 USA; 11 Goodman-Luskin Microbiome Center, University of California, Los Angeles, Los Angeles, CA 90095 USA

**Keywords:** Uterine endometrial carcinoma (UEC), Chimeric antigen receptor (CAR), Natural killer T (NKT) cell, Stem cell engineering, Allogeneic CAR-NKT cells, Mesothelin (MSLN), Allogeneic cell therapy, Off-the-shelf, Potent antitumor activity, Tumor microenvironment (TME), Tumor-associated macrophage (TAM), Multiple tumor targeting mechanism, Antigen escape.

## Abstract

**Background:**

Aggressive subtypes of uterine endometrial carcinoma (UEC) often result in mortality due to recurrence of disease with chemoresistant tumor cells surrounded by an immune suppressive microenvironment. Current CAR-T cell therapies have shown limited efficacy in solid tumors, largely constrained by poor tumor infiltration, immune suppression, and the logistical limitations of autologous cell production, which hinder broad patient access.

**Methods:**

In this study, we conducted comprehensive immunophenotyping of primary UEC patient samples and identified a therapeutic opportunity for CAR-engineered invariant natural killer T (CAR-NKT) cells capable of targeting both tumor cells and the immunosuppressive TME. Using a hematopoietic stem and progenitor cell (HSPC) engineering platform coupled with ex vivo differentiation culture, we generated allogeneic mesothelin-targeting CAR-NKT cells (^Allo^MCAR-NKT) with high purity and yield.

**Results:**

^Allo^MCAR-NKT cells exhibited potent cytotoxic activity against UEC tumor cells and CD1d⁺ tumor-associated macrophages (TAMs) and myeloid-derived suppressor cells (MDSCs). Importantly, compared to conventional CAR-T cells, ^Allo^MCAR-NKT cells demonstrated an improved safety profile, showing no evidence of graft-versus-host disease (GvHD) and minimal cytokine release syndrome (CRS)-related toxicity.

**Conclusion:**

These findings highlight the potential of ^Allo^MCAR-NKT cells as a safe and effective off-the-shelf cellular immunotherapy for the treatment of UEC and potentially other solid tumors characterized by an immunosuppressive microenvironment.

**Supplementary Information:**

The online version contains supplementary material available at 10.1186/s40164-026-00746-8.

## Background

Carcinomas of the endometrium arising from the inner lining of the uterus pose a significant clinical challenge as the 5th leading cause of cancer-related death in women and is a malignancy for which survival has consistently decreased over the last 40 years. Since the 2000’s, incidence of uterine cancers has steadily increased by about 1% annually [1]. The spectrum of endometrial cancers includes endometrioid adenocarcinomas, which are often detected at early stages and, in low-risk patients, can frequently be managed with non-invasive surgery alone. In contrast, high-risk histological subtypes, including uterine papillary serous carcinomas (UPSCs), clear cell adenocarcinomas, undifferentiated carcinomas, mixed adenocarcinomas, and carcinosarcomas, tend to present at advanced stages and account for a disproportionate share of mortality [2, 3]. Among these, UPSCs are particularly aggressive, presenting with advanced-stage disease in approximately half of cases [2]. Although they comprise about 10% of all endometrial cancers, they contribute to 39% of deaths [2]. Patients diagnosed at stage III/IV have only a 33% 5-year survival rate despite receiving standard of care treatments including radical surgery in conjunction with platinum-based chemotherapy and/or radiation [2, 4]. Contributing to these tumors’ aggressive biologic behavior is the presence of p53 mutations in about 90% of uterine serous carcinoma cases coupled with an immunosuppressive microenvironment [5, 6]. While checkpoint inhibition has expanded treatment options for some mismatch repair-deficient uterine endometrial carcinomas (UECs), aggressive UPSCs are typically mismatch repair-proficient rendering this therapeutic option less effective [7]. Additionally, in the recurrent and advanced setting, a combination of lenvatinib and pembrolizumab is used to treat these tumors, though response rates are low and often not durable [8–10]. Antibody drug conjugates (ADCs) targeting HER2 have been utilized for the treatment of recurrent tumors, with reported median progression free survival (PFS) of approximately 11 months [11]. The persistent UEC treatment challenges compounded by their aggressive nature and harrowing clinical outcomes emphasizes demand for novel research dedicated to overcoming the current treatment limitations.

Recognition of this critical research gap has prompted an increased interest in cell-based immunotherapies. One such approach is the use of chimeric antigen receptor (CAR)- engineered T (CAR-T) cell therapy, which entails genetic engineering T cells to express receptors capable of recognizing and killing tumor cells [12–14]. CAR-T cell therapy has demonstrated clinical success in treating hematological malignancies, and is now being investigated in solid tumors [15]. While CAR-T cell therapy has not yet received FDA approval for the treatment of solid tumors, several Phase I/II clinical trials are actively recruiting patients (NCT06215950, NCT06241456, NCT06658951, NCT04503278, NCT04627740). These trials aim to investigate the safety and efficacy of CAR-T cell construct in solid tumor indications, including those of uterine or endometrial origin. While promising, the application of CAR-T cells in the solid tumor setting is impacted by several inherent limitations and multifaceted challenges. For example, UECs often exhibit tumor heterogeneity leading to variable antigen expression and potential antigen escape [16]. The tumor microenvironement (TME) poses another challenge as it contains several immunosuppressive cell populations such as regulatory T cells (Tregs), myeloid-derived suppressor cells (MDSCs), and tumor-associated macrophages (TAMs) [17–21]. These cells secrete inhibitory cytokines leading to suppressed CAR-T function and potentially CAR-T cell exhaustion. Beyond cellular components, the TME presents physical and metabolic barriers including hypoxia and acidosis, which negatively effect CAR-T cell proliferation and metabolism [22]. Rapid tumor cells proliferation created metabolic competition, further starving and impairing CAR-T cell activity. Furthermore CAR-T cell therapy can result in systemic toxicities, most notably cytokine release syndrome (CRS), a potentially severe inflammatory response caused by the rapid release of cytokines by activated CAR-T cells [23]. Lastly, the complex and expensive CAR-T cell manufacturing process limits the scalability and widespread accessibility of this treatment [24]. These multifaceted limitations inherent to CAR-T cell therapy have prompted the development of alternate cell-based immunotherapy platforms that may be better poised to tackle the complexities of UECs and other solid malignancies.

CAR-NKT cells represent a novel and promising approach in cellular immunotherapy, characterized by potent antitumor activity, multiple tumor cell killing mechanisms, efficient trafficking and infiltration into solid tumors, and a unique ability to modulate the immunosuppressive TME [25–31]. Critically, because the invariant NKT TCR recognizes the non-polymorphic CD1d molecule rather than classical MHC antigens, CAR-NKT cells do not induce graft-versus-host disease (GvHD), enabling their use as off-the-shelf allogeneic cell products [32–36]. However, the low frequency of NKT cells in human peripheral blood (0.001–1%) presents a major barrier to the scalable generation of allogeneic CAR-NKT cells for clinical application [37, 38].

To overcome this limitation, we previously established a robust platform to generate allogeneic CAR-NKT cells using lentiviral engineering of human hematopoietic stem and progenitor cells (HSPCs) followed by ex vivo differentiation [39]. In the current study, we build upon this platform to develop allogeneic mesothelin-targeting CAR-NKT (^Allo^MCAR-NKT) cells for the treatment of UEC. We performed a comprehensive evaluation of ^Allo^MCAR-NKT cells using a combination of in vitro functional assays, in vivo xenograft models, primary UEC patient samples, patient-derived tumor cell lines, and single-cell RNA sequencing. Key aspects analyzed included the manufacturing efficiency, phenotypic and functional characteristics, antitumor efficacy, and safety profile of ^Allo^MCAR-NKT cells. This study provides strong preclinical evidence supporting the translational potential of ^Allo^MCAR-NKT cells as an effective and safe off-the-shelf immunotherapy for UEC.

## Methods

### Lentiviral vectors

A parental lentivector, pMNDW, was utilized to construct the lentiviral vectors employed in this study [40, 41]. The 2 A sequences derived from foot-and-mouth disease virus (F2A), porcine teschovirus-1 (P2A), and thosea asigna virus (T2A) were used to link the inserted genes to achieve co-expression. The Lenti/iNKT-MCAR vector was generated by inserting into the pMNDW parental backbone a synthetic tricistronic gene encoding human iNKT TCRα-F2A-iNKT TCRβ-P2A-MCAR (MCAR denotes an MSLN-specific CAR). The Lenti/iNKT-MCAR-IL-15 vector was generated by inserting into the pMNDW parental backbone a synthetic tetracistronic gene encoding human iNKT TCRα-F2A-iNKT TCRβ-P2A-MCAR-T2A-IL-15 (IL-15 represents the secreted form of human interleukin-15). The Lenti/MCAR vector was constructed by inserting a synthetic gene encoding MCAR into pMNDW. The Lenti/MCAR-IL-15 vector was constructed by inserting a synthetic bicictronic gene encoding MCAR-P2A-IL-15 into pMNDW. The Lenti/FG vector was generated by inserting a synthetic bicistronic gene encoding Fluc-P2A-EGFP into the pMNDW backbone. The Lenti/MSLN vector was constructed by inserting a synthetic gene encoding human MSLN into pMNDW. All synthetic gene fragments were obtained from GenScript (Piscataway, NJ, USA) and Integrated DNA Technologies (IDT; Coralville, IA, USA). Lentiviral particles were generated utilizing HEK 293 T cells by employing a standardized transfection procedure with the Trans-IT-Lenti Transfection Reagent (Mirus Bio) [40, 41]. Subsequently, a concentration protocol was applied using Amicon TM Ultra Centrifugal Filter Units in accordance with the manufacturer’s specifications (MilliporeSigma).

### Stable cell lines

Human UEC cell line HEC1B (cat. no. HTB-113) was purchased from the American Type Culture Collection (ATCC). OVCAR8 human ovarian cancer (OC) cell lines were generously provided by the Division of Cancer Treatment and Diagnosis (DCTD) Tumor Repository at the National Institutes of Health (NIH). To establish stable tumor cell lines that overexpress firefly luciferase and green fluorescent protein dual reporters (FG), the parental tumor cell lines were transduced with lentiviral vectors carrying the specific genes of interest (i.e., Lenti/FG). 72 h after lentiviral transduction, the cells underwent flow cytometry sorting to isolate the genetically modified cells (as identified as GFP^+^ cells) necessary for creating stable cell lines. The artificial antigen presenting cell (aAPC) line was generated by engineering the K562 human chronic myelogenous leukemia cell line (ATCC, cat. no. CCL-243) to overexpress human CD80/CD83/CD86/41BBL co-stimulatory receptors [39]. The aAPC-MSLN cell lines were generated by further engineering the parental aAPC line to overexpress human MSLN.

### Human CD34^+^ hematopoietic stem and progenitor cells (HSPCs) and periphery blood mononuclear cells (PBMCs)

Purified human CD34^+^ HSPCs derived from cord blood (CB) were purchased from HemaCare. Healthy donor PBMCs were provided by the UCLA/CFAR Virology Core Laboratory without identification information under federal and state regulations. Upon receipt, both HSPCs and PBMCs were promptly aliquoted and cryopreserved in liquid nitrogen for subsequent experimental use.

### Primary patient sample collection and processing

Approval from the UCLA Office of the Human Research Protection Program (IRB#10–0727, IRB#20–1659) was granted for the procedures outlined in this study. All human specimens (ascites or solid tumor) were collected from patients who provided informed consent. Clinical information was obtained from the medical records. The study population consisted of patients diagnosed with uterine papillary serous carcinomas at chemonaive or recurrent disease states.

Fresh ascites specimens were collected and immediately processed in the laboratory. Tumor cells were isolated through centrifugation, resuspended in ice cold cryopreservation buffer (FBS + 10% DMSO), and viably cryopreserved in vapor phase liquid nitrogen for long term storage. Solid tumor specimens were collected fresh and brought back to the laboratory for processing. Specimens were minced into < 5 mm pieces prior to enzymatic (RPMI, collagenase, dispase, DNase) and mechanical (37˚C, 300 rpm shaking incubator) dissociation. The dissociated tumor cells were subjected to RBC lysis using the manufacturers protocol (BioLegend), strained through a 100 μm filter, then resuspended in ice cold cryopreservation buffer (FBS + 10%DMSO) prior to storage in vapor phase liquid nitrogen.

### Patient-derived tumor (PDT) cell line

A primary ascites specimen was obtained from a consented patient diagnosed with high grade uterine papillary serous endometrial carcinoma. Tumor cells were isolated from the ascites by centrifugation and plated in vitro with cell culture media (RPMI/10% FBS; rock inhibitor [Y-27632 2HCL, Selleckchem] supplemented for the first passage only). Tumor cells were serially passaged in RPMI/10% FBS in vitro for five passages. Genomic DNA was extracted from expanded cells and analyzed by short tandem repeat (STR) and single nucleotide polymorphism (SNP) to validate the relatedness of the PDT cell line to the primary parental ascites sample. STR and SNP results confirmed high relatedness. PDT cells were viably cryopreserved in buffer (90% FBS + 10% DMSO) and stored in vapor phase liquid nitrogen for future experimental use. The PDT cell line was further engineered using a Lenti/FG lentiviral vector and subsequently sorted for GFP⁺ cells for use in downstream experiments.

### Enzyme-linked immunosorbent cytokine assays (ELISAs)

The ELISAs for measuring cytokines or biomarkers were conducted according to a standard protocol provided by BD Biosciences. Samples were collected and analyzed to quantify cytokines or biomarkers. The capture and biotinylated antibodies used for cytokine detection were sourced from BD Biosciences, while the streptavidin-HRP conjugate was obtained from Invitrogen. Human and mouse cytokine standards were purchased from eBioscience, and the Tetramethylbenzidine (TMB) substrate was acquired from Thermo Scientific (cat. no. PI34021). Absorbance of the samples was measured at 450 nm using an Infinite M1000 microplate reader (Tecan).

### Immunohistochemistry (IHC) staining

Immunohistochemistry was used to detect mesothelin and PAX8 in histologic sections of patient effusion samples. The primary antibodies used to detect tumor cells were anti-mesothelin (SP74, Abcam, 1:250) anti-Pax8 (MRQ-50, Cell Marque, 1:500). The secondary antibodies used were biotinylated goat anti-rabbit (Jackson Immunoresearch, 1:1000) and biotinylated rabbit anti-mouse (Jackson Immunoresearch, 1:1000). The tertiary antibody used was streptavidin-conjugated horseradish peroxidase (Jackson Immunoresearch, 1:1000). 3,3’-diaminobenzidine (DAB) chromagen (HK130-5 K, Biogenex) was used for detection.

### Generation of HSPC-engineered ^Allo/15^MCAR-NKT cells

^Allo/15^MCAR-NKT cells were generated by differentiating gene engineered human cord blood CD34^+^ HSPCs in a 5-stage clinically guided Ex Vivo HSPC-Derived CAR-NKT Cell Culture method. The complete methodology and step-by-step protocols have been described in detail in previously published studies [39, 42–44]. Here, we provide a summary of the key steps involved in the culture and generation of ^Allo/15^MCAR-NKT cells.

At Stage 0, the frozen stock of human CD34^+^ HSPCs was thawed and cultured in T cell X-VIVO 15 Serum-Free Hematopoietic Stem Cell Medium supplemented with human Flt3L (50 ng/ml), SCF (50 ng/ml), TPO (50 ng/ml), and IL-3 (20 ng/ml) for 24 h. Lentiviral transduction was subsequently carried out for an additional 24 h using the Lenti/iNKT-MCAR-IL-15 or Lenti/iNKT-MCAR vector.

At Stage 1, gene-engineered HSPCs harvested were cultured in the feeder-free StemSpan™ SFEM II Medium supplemented with StemSpan™ Lymphoid Progenitor Expansion Supplement for 14 days. HSPCs were cultured in CELLSTAR^®^24-well Cell Culture Nontreated Multiwell Plates (VWR, cat. no. 82050-892). StemSpan™ Lymphoid Differentiation Coating Material (500 µl/well, diluted to a final concentration of 1X from a stock dilution of 100X) was applied to the plates and left for 2 h at room temperature or overnight at 4°C. Subsequently, 500 µl of the transfected CD34^+^ HSPC suspension, with a density of 2 × 10^4^ cells/ml, was added to each pre-coated well. Half of the medium in each well was removed and replaced with fresh medium twice per week.

At Stage 2, the Stage 1 cells were harvested and cultured in the feeder-free StemSpan™ SFEM II Medium supplemented with StemSpan™ Lymphoid Progenitor Maturation Supplement for ~ 7 days. StemSpan™ Lymphoid Differentiation Coating Material (1 ml/well, diluted to a final concentration of 1X) was applied to Non-Treated Falcon™ Polystyrene 6-well Microplates (Thermo Fisher Scientific, cat. no. 140675); 2 ml of the harvested Stage 1 cells, resuspended with a density of 1 × 10^5^ cells/ml, was added into each pre-coated well. The cell density was maintained at 1–2 × 10^6^ cells per well during the Stage 2 culturing. Cells were passaged 2–3 times per week with the addition of fresh medium for each passage.

At Stage 3, the Stage 2 cells were harvested and cultured in the feeder-free StemSpan™ SFEM II Medium supplemented with StemSpan™ Lymphoid Progenitor Maturation Supplement, CD3/CD28/CD2 T Cell Activator, and human recombinant IL-15 (20 ng/ml) for ~ 7 days. StemSpan™ Lymphoid Differentiation Coating Material (1 ml/well, diluted to a final concentration of 1X) was applied to Non-Treated Falcon™ Polystyrene 6-well Microplates (Thermo Fisher Scientific, cat. no. 08–772-49); 2 ml of the harvested Stage 2 cells, resuspended with a density of 5 × 10^5^ cells/ml, was added into each pre-coated well. The cell density was maintained at 1–2 × 10^6^ cells per well during the Stage 3 culturing. Cells were passaged 2–3 times per week with the addition of fresh medium for each passage.

At Stage 4, the Stage 3 cells were harvested and verified by flow cytometry to confirm their status as mature ^Allo15^MCAR-NKT cells or their derivatives; then the cells underwent expansion stage via an aAPC-based expansion. aAPC-MSLN cells were irradiated at 10,000 rads using a Rad Source RS-2000 X-Ray Irradiator (Rad Source Technologies). The Stage 3 mature ^Allo/U15^MCAR-NKT cells and derivatives were co-cultured with the irradiated aAPC-MSLN cells (with a ratio of 1:1). The cells were resuspended in expansion medium (the CTS™ OpTimizer™ T-Cell Expansion Serum Free Medium (Thermo Fisher Scientific), or the homemade C10 medium) supplemented with human IL-7 (10 ng/ml) and IL-15 (10 ng/ml) at a density of 0.5–1.5 × 10^6^ cells/ml; 2 ml cell suspension was seeded into each well of the Corning™ Costar™ Flat Bottom Cell Culture 6-well Plates. The cell density was maintained at 0.5–1.5 × 10^6^ cells/ml during the expansion stage. Cells were passaged 2–3 times per week with the addition of fresh medium for each passage. The expanded ^Allo/U15^MCAR-NKT cells were aliquoted and cryopreserved in CryoStor^®^ Cell Cryopreservation Media CS10 using a Thermo Scientific™ CryoMed™ Controlled-Rate Freezer 7450 (Thermo scientific) for stock.

### Generation of PBMC-derived conventional αβ T cells

PBMCs from healthy donors were used to generate conventional αβ T cells (hereafter referred to as T cells). T cell activation was achieved using one of two methods: (1) stimulation with Dynabead™ Human T-Activator CD3/CD28 (Thermo Fisher Scientific, Cat. No. 11131D) according to the manufacturer’s instructions, or (2) plate-bound activation. For the latter, non-treated 24-well tissue culture plates (Corning, Cat. No. 3738) were coated with Ultra-LEAF™ purified anti-human CD3 antibody (Clone OKT3, BioLegend, cat. no. 317325) at 1 µg/ml (500 µl/well) for 2 h at room temperature or overnight at 4 °C. PBMCs were then resuspended in C10 medium supplemented with 1 µg/ml Ultra-LEAF™ purified anti-human CD28 antibody (Clone CD28.2, BioLegend, cat. no. 302933) and 30 ng/ml IL-2, and seeded into the pre-coated plates at a density of 1 × 10^6^ cells/ml (1 ml/well). Following activation, cells were maintained in C10 medium supplemented with 20 ng/ml IL-2 and cultured for 2–3 weeks.

### Generation of MSLN-targeting CAR-engineered conventional αβ T (MCAR-T) cells

PBMCs from healthy donors were utilized to generate conventional MCAR-T cells. To produce these cells, non-treated tissue culture 24-well plates (Corning, cat. no. 3738) were coated with Ultra-LEAF™ Purified Anti-Human CD3 Antibody (Clone OKT3, BioLegend) at 1 µg/ml (500 µl/well), at room temperature for 2 h or at 4 °C overnight. PBMCs were resuspended in the C10 medium supplemented with 1 µg/ml Ultra-LEAF™ Purified Anti-Human CD28 Antibody (Clone CD28.2, BioLegend) and 30 ng/ml IL-2, followed by seeding in the pre-coated plates at 1 × 10^6^ cells/ml (1 ml/well). After 2 days, the cells were transduced with Lenti/MCAR viruses for a period of 24 h. Conventional MCAR-T cells were expanded for approximately two weeks in C10 medium, with CAR expression confirmed by flow cytometry, followed by cryopreservation for subsequent use. In this study, all MCAR-T cells used in the in vitro tumor cell killing assays and in vivo human cancer xenograft mouse models were normalized according to the proportion of CAR⁺ cells.

### Generation of PBMC-derived IL-15-enhanced MCAR-engineered NK (^PBMC15^MCAR-NK) cells

Healthy donor PBMCs were sorted with MACS via a Human NK Cell Isolation Kit (Miltenyi Biotech) to enrich NK cells, following the manufacturer’s instructions and previous studies [34]. The enriched NK cells were mixed with irradiated aAPCs at a ratio of 1:10, followed by culturing in C10 medium supplemented with 10 ng/ml IL-7 and IL-15. On day 3, NK cells were transduced with Lenti/MCAR-IL15 viruses for 24 h. The resulting ^PBMC15^MCAR-NK cells were expanded for about 1 week in C10 medium supplemented with 10 ng/ml IL-7 and IL-15. In this study, fresh-cultured ^PBMC15^MCAR-NK cells were utilized in the in vitro tumor cell killing assays.

### In vitro tumor cell killing assay

Human tumor cells (e.g., HEC1B-FG and HEC1B-MSLN-FG; 1 × 10^4^ cells per well in 96-well plate) were co-cultured with the indicated therapeutic cells (i.e., T, MCAR-T, and ^Allo/15^MCAR-NKT cells) in Corning 96-well clear bottom black plates for 24 h in C10 medium. The effector cell to target cell (E: T) ratio is indicated in the figure legends. At the end of culture, viable tumor cells were quantified by adding D-luciferin (150 µg/ml; Fisher Scientific, cat. no. 50–209-8110) to cell cultures, followed by the measurement of luciferase activity using an Infinite M1000 microplate reader (Tecan).

### In vitro serial tumor cell killing assay

1 × 10^4^ non-engineered tumor cells (e.g., PDT cells; referred to as stimulator cells) were co-cultured with 2 × 10^5^ therapeutic cells in a Corning 96-well clear bottom black plate within C10 medium. Cultures were supplemented with a dose of 1 × 10^4^ stimulator cells every 2 days. Stimulator cells were then substituted with 1 × 10^4^ of FG-engineered tumor cells (e.g., PDT-FG cells; referred to as indicator cells) 24 h prior to luminescent read-out of tumor killing. On the day of imaging, remaining live indicator cells were quantified through addition of 100 µL of D-Luciferin (150 µg/ml) with subsequent readout using an Infinite M1000 microplate reader (Tecan) to measure luciferase activity from residual indicator cells.

### In vitro assays using primary UEC patient samples

In one assay, the primary UEC patient samples were analyzed for tumor cell phenotype and the TME composition using flow cytometry. UEC tumor cells identified as CD45^−^CD31^−^FAP (fibroblast activation protein)^−^ cells [45, 46], T cells were identified as CD45^high^CD3^+^ cells, CD4 T cells were identified as CD45^high^CD3^+^CD4^+^ T cells, CD8 T cells were identified as CD45^high^CD3^+^CD8^+^ T cells, B cells were identified as CD45^high^CD19^+^ or CD45^high^CD20^+^ cells, NK cells were identified as CD45^high^CD56^+^CD3^−^ cells, monocytes were identified as CD45^high^CD11b^+^CD14^+^ cells, TAMs were identified as HLA-DR^high^CD206^high^ monocytes, and MDSCs were identified as HLA-DR^low^CD206^low^ monocytes. Surface expression of CAR targets and NK ligands on tumor or/and immune cells were also analyzed using flow cytometry.

In another assay, the primary UEC patient samples were used to study tumor cell killing by ^Allo/15^MCAR-NKT or conventional MCAR-T cells. Tumor cells were pre-sorted using a Human Tumor Cell Isolation Kit (Miltenyi Biotec, cat. no. 130-108-339), followed by co-culturing with various therapeutic cells (E: T ratio 1:1) in C10 medium in Corning 96-well Round Bottom Cell Culture plates for 24 h. At the end of culture, cells were collected and live UEC tumor cells (identified as CD45^−^CD3^−^6B11^−^ cells) was analyzed using flow cytometry. A total of 6 primary UEC patient samples were included in this assay.

In another assay, the primary UEC patient samples were used to study the TME targeting by ^Allo/15^MCAR-NKT cells or conventional MCAR-T cells. Patient samples were directly co-cultured with various therapeutic cells (ratio 1:1) in C10 medium in Corning 96-well Round Bottom Cell Culture plates for 24 h. At the end of culture, cells were collected, and the TME targeting was assessed using flow cytometry by quantifying live human TAM (identified as 6B11^−^MCAR^−^CD45^+^CD14^+^CD11b^+^HLA-DR^high^CD206^high^ cells), MDSCs (identified as 6B11^−^MCAR^−^CD45^+^CD14^+^CD11b^+^HLA-DR^low^CD206^low^ cells), T cells (identified as 6B11^−^MCAR^−^CD45^+^CD3^+^ cells), B cells (identified as 6B11^−^MCAR^−^CD45^+^CD3^−^CD19^+^ cells or 6B11^−^MCAR^−^CD45^+^CD3^−^CD20^+^ cells), and NK cells (identified as 6B11^−^MCAR^−^CD45^+^CD3^−^CD56^+^ cells). To investigate the CD1d-mediated killing mechanism, patient samples were directly co-cultured with ^Allo/15^MCAR-NKT cells (ratio 2:1), and 10 µg/ml LEAF™ purified anti-human CD1d antibody (Clone 51.1, Biolegend) or LEAF™ purified mouse IgG2b k isotype control antibody (Clone MG2b-57, Biolegend) was added to the co-cultures. A total of 6 primary UEC patient samples were included in this assay.

### In vivo bioluminescence live animal imaging (BLI)

BLI was conducted using the Spectral Advanced Molecular Imaging (AMI) HTX system (Spectral Instrument Imaging). Live animal images were captured 5 min after intraperitoneal (i.p.) administration of D-Luciferin, with doses of 1 mg/mouse for tumor cell (e.g., HEC1B-FG cell) visualization. For tissue imaging, experimental mice received an i.p. injection of D-Luciferin with doses of 10 mg/mouse. Mice were then euthanized, and tissues were collected for BLI. The imaging data were processed and analyzed using AURA imaging software (Spectral Instrument Imaging, version 3.2.0).

### In vivo antitumor efficacy study: PDT-FG human UEC xenograft NSG mouse model

On Day 0, NSG mice received i.p. injection of human UEC tumor cells (PDT-FG, 5 × 10^6^ cells per mouse). On Day 10, the experimental mice received i.p. injection of vehicle (100 µl PBS per mouse), ^Allo/15^MCAR-NKT cells (10 × 10^6^ MCAR^+^ cells in 100 µl PBS per mouse), or control MCAR-T cells (10 × 10^6^ MCAR^+^ cells in 100 µl PBS per mouse). Over the experiment, mice were monitored for survival and their tumor load were measured using BLI. At the study endpoint, experimental mice were euthanized, and survival data were collected to generate Kaplan–Meier survival curves. All in vivo experiments in this study were independently repeated at least three times.

### In vivo antitumor efficacy study: HEC1B(-MSLN)-FG human UEC xenograft NSG mouse model

On Day 0, NSG mice received i.p. injection of human UEC tumor cells (HEC1B-FG or HEC1B-MSLN-FG, 5 × 10^5^ cells per mouse). On Day 9, the experimental mice received i.p. injection of vehicle (100 µl PBS per mouse), ^Allo/15^MCAR-NKT cells (3 × 10^6^ or 10 × 10^6^ MCAR^+^ cells in 100 µl PBS per mouse), or control MCAR-T cells (3 × 10^6^ or 10 × 10^6^ MCAR^+^ cells in 100 µl PBS per mouse). Over the experiment, mice were monitored for survival and their tumor load were measured using BLI. At the study endpoint, experimental mice were euthanized, and survival data were collected to generate Kaplan–Meier survival curves.

### In vivo antitumor efficacy study: OVCAR8-FG human OC xenograft NSG mouse model

On Day 0, NSG mice received i.p. injection of human OC tumor cells (OVCAR8-FG, 5 × 10^5^ cells per mouse). On Day 4, the experimental mice received i.p. injection of vehicle (100 µl PBS per mouse), ^Allo/15^MCAR-NKT cells (3 × 10^6^ MCAR^+^ cells in 100 µl PBS per mouse), or control MCAR-T cells (3 × 10^6^ MCAR^+^ cells in 100 µl PBS per mouse). Over the experiment, mice were monitored for survival and their tumor load were measured using BLI. At the study endpoint, experimental mice were euthanized, and survival data were collected to generate Kaplan–Meier survival curves.

### In vivo graft-versus-host disease (GvHD) study

On Day 0, NSG mice received intravenously (i.v.) injection of ^Allo15^MCAR-NKT cells (10 × 10^6^ MCAR^+^ cells in 100 µl PBS per mouse), or conventional MCAR-T cells (10 × 10^6^ MCAR^+^ cells in 100 µl PBS per mouse). Over the experiment, mice were monitored for survival and their body weight and GvHD score were measured. A score ranging from 0 to 2 was assigned for each clinical GvHD sign, which includes body weight, activity, posture, skin thickening, diarrhea, and dishevelment [35].

### In vivo cytokine release syndrome (CRS) study

On Day 0, NSG mice received i.p. injection of a high dose of HEC1B-MSLN-FG tumor cells (10 × 10^6^ cells per mouse). On Day 10, the experimental mice received i.p. injection of vehicle (100 µl PBS per mouse), ^Allo15^MCAR-NKT cells (10 × 10^6^ CAR^+^ cells in 100 µl PBS per mouse), or conventional MCAR-T cells (10 × 10^6^ CAR^+^ cells in 100 µl PBS per mouse). On Days 13, blood and peritoneal fluid samples were collected from the experimental mice, and the mouse IL-6 and SAA-3 were measured using ELISA. A Mouse SAA-3 ELISA Kit (Millipore Sigma) was used to measure SAA-3, following the manufacturer’s instructions.

### Single cell RNA sequencing (scRNA-seq)

Freshly collected samples were immediately delivered to the UCLA TCGB Core for library construction and scRNA-seq. Cells were quantified using a Cell Countess II automated cell counter (Invitrogen/Thermo Fisher Scientific). A total of 10,000 cells from each experimental group were loaded on the Chromium platform (10X Genomics), and libraries were constructed using the Chromium Next GEM Single Cell 3’ Kit and the Chromium Next GEMChip G Single.

Cell Kit (10X Genomics), according to themanufacturer’s instructions. Library quality was assessed using the D1000 ScreenTape on a 4200 TapeStation System (Agilent Technologies). Libraries were sequenced on an Illumina NovaSeq using the NovaSeq S4 Reagent Kit (100 cycles; Illumina). AddModuleScore was used to calculate module scores of each list of gene signatures, and FeaturePlot function was used to visualize the expression of each signature in the UMAP plots [47, 48]. GSEA and gseaNb were used to calculate the enrichment score of effector, memory, and cytotoxic gene signatures in the indicated cell types.

### Histological analysis of mouse tissues

Mouse tissues were collected from mice and fixed in 10% Neutral Buffered Formalin for 72 h, then embedded in paraffin. Tissues were sectioned to 4 μm thickness and stained with Hematoxylin and Eosin (H&E) by the UCLA Translational Pathology Core Laboratory, per the Core’s standard protocols. The sections were imaged on an Olympus BX51 upright microscope equipped with an Optronics Macrofire CCD camera (AU Optronics). The images were analyzed using Optronics PictureFrame software (AU Optronics).

### Statistics

Graphpad Prism 9 software (Graphpad) was used for statistical data analysis. Student’s two-tailed t test was used for pairwise comparisons. Ordinary 1-way ANOVA followed by Tukey’s or Dunnett’s multiple comparisons test was used for multiple comparisons. Log rank (Mantel-Cox) test adjusted for multiple comparisons was used for Meier survival curves analysis. Data are presented as the mean ± SEM, unless otherwise indicated. In all figures and figure legends, “n” represents the number of samples or animals used in the indicated experiments. A P value of less than 0.05 was considered significant. ns, not significant.

## Results

### Primary UEC patient sample profiling highlights the unique therapeutic potential of CAR-NKT cells

**Fig. 1:**
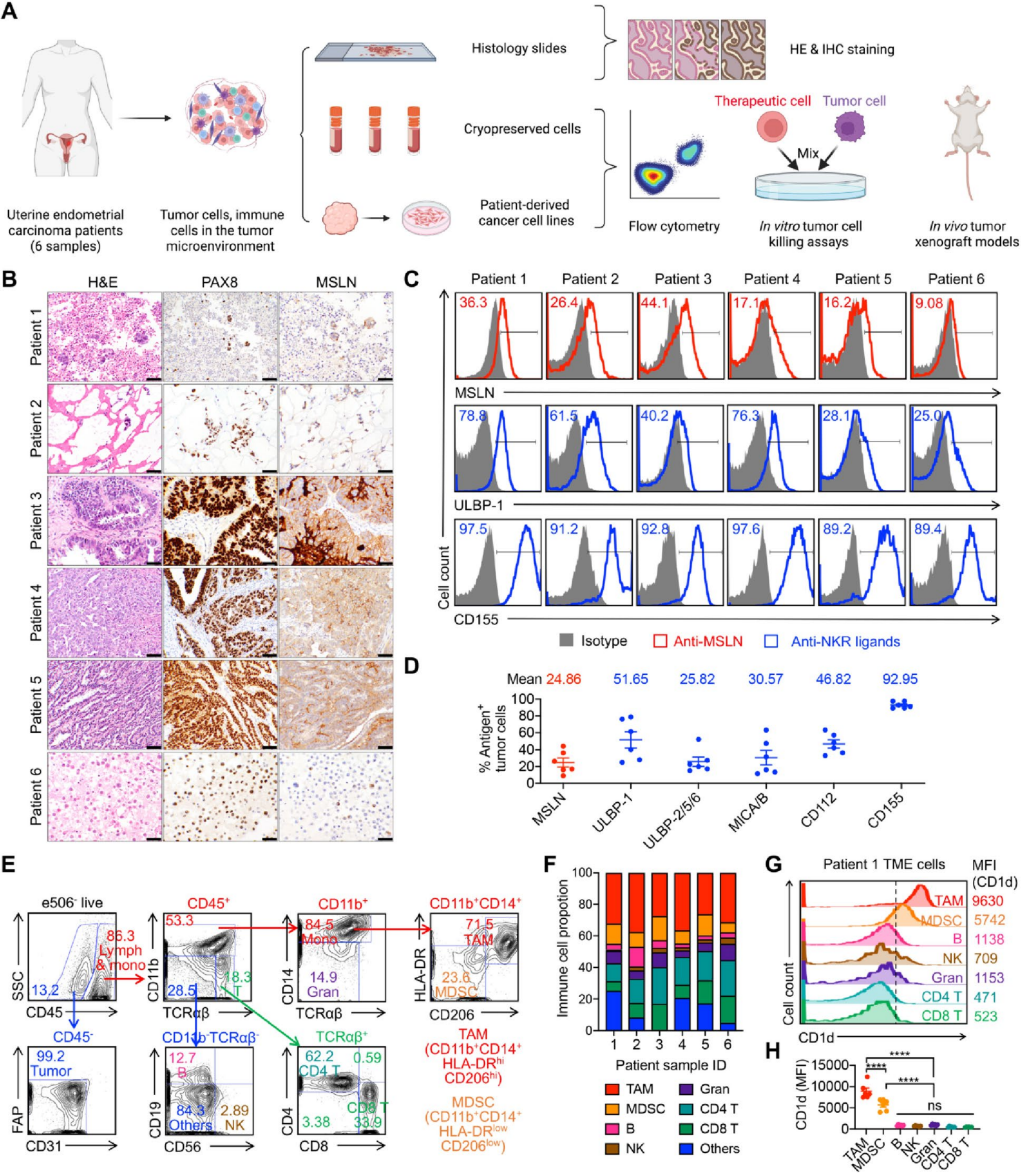
Primary uterine endometrial carcinoma (UEC) patient sample profiling highlights the unique therapeutic potential of CAR-NKT cells. **A** Schematics showing the comprehensive profiling of UEC patient samples using a series of experimental assays. H&E, Hematoxylin and Eosin staining; IHC, Immunohistochemistry. **B** H&E and IHC staining of representative histologic sections of primary UEC patients’ samples. The H&E stains show a pleomorphic neoplasm with high nuclear to cytoplasmic ratios and prominent nuclei. All samples show positive nuclear staining for PAX8 as well as cytoplasmic staining for mesothelin (MSLN). Scale bar represents 100 μm (20x objective). **C** FACS plots showing the detection of CAR target (i.e., MSLN) and NKR ligands (i.e., ULBP-1 and CD155) on primary patient-derived tumor cells. **D** Quantification of (**C**) (*n* = 6). **E** FACS gating strategies for tumor cells and immune cells in the UEC TME. Gran, granulocyte; lymph, lymphocyte; mono, monocyte; TAM, tumor-associated macrophage; MDSC, myeloid-derived suppressor cell; FAP, fibroblast activation protein; NK, natural killer cell. **F** TME immune cell proportions of primary UEC samples (*n* = 6). **G** and **H** FACS measurements of CD1d expression on the indicated immune cells in the UEC TME. **G** Representative FACS plots. **H** Quantification of (**G**) (*n* = 6). Data are presented as the mean ± SEM. ns, not significant, ****p < 0.0001 by one-way ANOVA (**H**)

We first conducted a comprehensive immunoprofiling of 6 primary UEC patient samples (Table S1), analyzing both tumor cells and the TME using hematoxylin and eosin (H&E) staining, immunohistochemistry (IHC), and flow cytometry (Fig. 1A). The goal was to identify actionable tumor-associated markers for the rational design of targeted cellular immunotherapies.

H&E staining revealed pleomorphic neoplastic cells characterized by high nuclear-to-cytoplasmic ratios and prominent nucleoli, consistent with high-grade malignancy (Fig. 1B). IHC analyses showed uniform nuclear positivity for PAX8 across all patient samples, confirming the Müllerian epithelial origin of the tumor cells (Fig. 1B) [49]. Additionally, strong cytoplasmic staining for mesothelin (MSLN) was observed, supporting its relevance as a potential target antigen for CAR-based therapies (Fig. 1B) [50, 51]. Notably, MSLN expression varied among patients, indicating intertumoral heterogeneity, a common feature of UEC (Fig. 1B) [52]. Flow cytometry confirmed MSLN expression and further revealed that UEC tumor cells expressed high levels of natural killer receptor (NKR) ligands, including ULBP family members and MICA/B (ligands for NKG2D), as well as CD112 and CD155 (ligands for DNAM-1) (Fig. 1B and D and S1A). Additionally, analysis of a publicly available patient cohort dataset of 175 UEC cases revealed that tumor cells from 7 patients exhibited high *MSLN* expression, while 114 patients showed medium expression (Figure S1B). Thus, approximately 69% of patients displayed medium-to-high *MSLN* levels, supporting MSLN as a promising therapeutic target for UEC. Overall, these findings suggest that UEC tumor cells are susceptible to both CAR-mediated and NKR-mediated cytotoxic mechanisms.

To further define the immunosuppressive landscape of UEC, we used flow cytometry to analyze immune cell populations within the TME. In addition to granulocytes and lymphocytes (CD4 T, CD8 T, B, and NK cells), we observed a substantial population of tumor-associated macrophages (TAMs) and myeloid-derived suppressor cells (MDSCs), both of which are known to suppress antitumor immunity and contribute to resistance to immunotherapy (Fig. 1E and F) [53–56]. Importantly, these immunosuppressive populations expressed significantly higher levels of CD1d, a non-polymorphic MHC class I-like molecule, compared to other immune cell subsets. This makes them amenable to targeting by invariant NKT cells through TCR/CD1d interactions (Figs. 1G, H, and S2).

In summary, the immunophenotypic characterization of primary UEC samples revealed a dual opportunity for CAR-NKT cell therapy: (1) direct targeting of tumor cells through both CAR and NKR-mediated mechanisms, and (2) modulation of the immunosuppressive microenvironment via TCR/CD1d-dependent elimination of TAMs and MDSCs. These findings provide a strong rationale for the development of CAR-NKT cell-based immunotherapies as a promising, multifaceted approach for treating UEC.

### Allogeneic MCAR-NKT cells can be generated from stem cell engineering and a clinically guided culture method with high purity and high yield

**Fig. 2:**
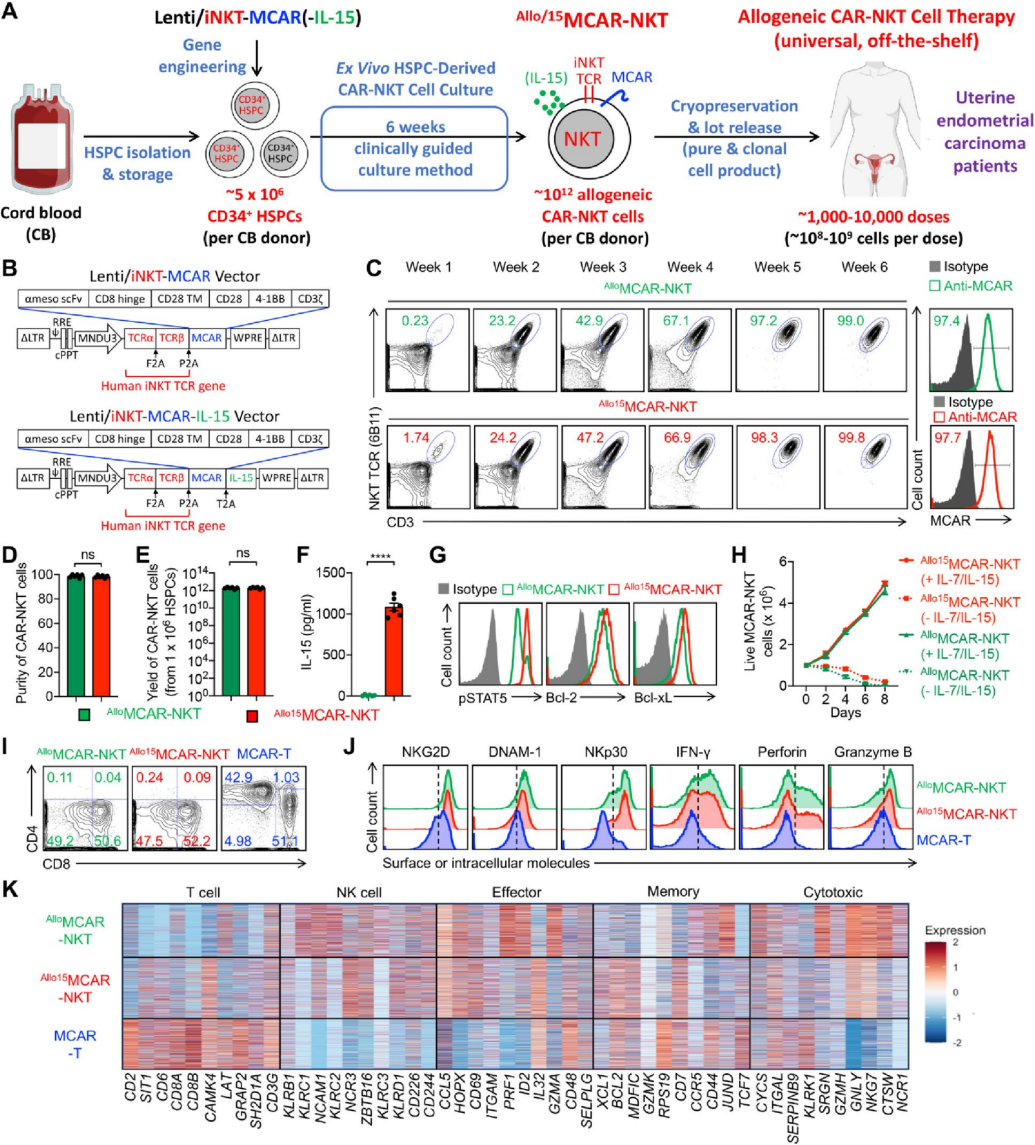
Allogeneic MCAR-NKT cells can be generated from stem cell engineering and a clinically guided culture method with high purity, yield, and potent cytotoxicity. **A** Schematics showing the development of a human hematopoietic stem and progenitor cell (HSPC)-engineered allogeneic MSLN-targeting CAR-armed NKT (^Allo^MCAR-NKT) cell product, as well as their IL-15-enhanced derivative (denoted as ^Allo15^MCAR-NKT cells), for treating uterine endometrial carcinoma (UEC). Lenti/iNKT-MCAR-(IL-15), lentiviral vector encoding a pair of human iNKT TCR α and β chains, an MSLN-targeting CAR, with or without a human soluble IL-15. **B** Schematics showing the design of the indicated lentivectors. iNKT, a pair of human iNKT TCR α and β chains; MCAR, MSLN-targeting CAR; LTR, long terminal repeats; MNDU3, internal promoter derived from the MND retroviral LTR U3 region; Ψ, packaging sequence; RRE, rev-responsive element; cPPT, central polypurine tract; WPRE, woodchuck hepatitis virus posttranscriptional regulatory element. **C** FACS monitoring of the generation of ^Allo/15^MCAR-NKT cells over the 6-week culture. A 6B11 monoclonal antibody was used to stain iNKT TCR. **D** Purity of ^Allo/15^MCAR-NKT cells (identified as iNKT TCR^+^CD3^+^MCAR^+^ cells; *n* = 6; n indicates different CB donors). **E** Yield of ^Allo/15^MCAR-NKT cells (*n* = 6; n indicates different CB donors). **F** ELISA analyses of IL-15 production by ^Allo/15^MCAR-NKT cells (*n* = 6). **G** FACS detection of IL-15-related biomarker expression in ^Allo/15^MCAR-NKT cells. **H** In vitro dysregulated growth assay. ^Allo/15^MCAR-NKT cells were cultured in vitro with/out addition of IL-7/IL-15, followed by quantification of live cells over time (*n* = 4). **I** FACS detection of CD4/CD8 co-receptor expression on ^Allo/15^MCAR-NKT cells. Healthy donor peripheral blood mononuclear cell (PBMC)-derived conventional MCAR-engineered T (MCAR-T) cells were included as a control. **J** FACS detection of NK receptor (NKR) expression, as well as intracellular cytokine and cytotoxic molecule production of ^Allo/15^MCAR-NKT and conventional MCAR-T cells. **K** Single cell RNA sequencing (scRNA-seq) analysis of ^Allo/15^MCAR-NKT cells. Heatmap showing the expression of representative signature genes. Each column indicates an individual gene. Each row indicates an individual cell. Representative of 1 (**K**) and > 6 (**A**–**J**) experiments. Data are presented as the mean ± SEM. ns, not significant, ****p < 0.0001 by Student’s *t* test (**D**–**F**)

We aimed to develop a new therapeutic approach targeting UEC by leveraging human HSPC gene engineering in combination with an established ex vivo HSPC differentiation method [39]. We previously utilized this platform to generate allogeneic BCMA-targeting and CD33-targeting CAR-NKT cells, achieving high yield and purity [39, 57]. Given the high expression of MSLN on UEC tumor cells, we extended this platform to develop MSLN-targeting CAR-NKT (MCAR-NKT) cells for UEC treatment (Fig. 2A) [43].

Human cord blood (CB)-derived CD34⁺ HSPCs were transduced with either a Lenti/iNKT-MCAR vector or a Lenti/iNKT-MCAR-IL-15 vector (Fig. 2A and B). Both lentiviral constructs encoded the invariant NKT TCR α and β chains, previously cloned from healthy donor NKT cells and used in both autologous and allogeneic HSPC-engineered NKT cell therapies [40, 41]. Additionally, both vectors carried a third-generation MSLN-targeting CAR construct consisting of the clinically validated SS1 anti-MSLN single-chain variable fragment (scFv) fused to a CD28–4-1BB–CD3ζ intracellular signaling domain [58, 59]. The IL-15–engineered vector additionally encoded a human secreted IL-15, which has been shown to enhance the persistence and antitumor efficacy of CAR-NKT cells in both preclinical and clinical settings [34, 39, 57, 60, 61].

Following lentiviral transduction, engineered HSPCs were subjected to a 6-week ex vivo culture protocol to generate allogeneic CAR-NKT cells, with or without IL-15 enhancement, named ^Allo15^MCAR-NKT and ^Allo^MCAR-NKT cells, respectively (Fig. 2B and C). Flow cytometry was used to monitor CAR-NKT cell expansion and differentiation during culture (Fig. 2C). CAR-NKT cells expanded from approximately 1% at week 1, to 20% at week 2, 40–50% at week 3, 60–70% at week 4, and exceeded 97% purity by week 5 (Fig. 2C). Importantly, the final product demonstrated high phenotypic purity (> 97%), with nearly all NKT cells co-expressing the CAR due to co-integration of iNKT TCR and CAR in the same lentivector and selective expansion of NKT cells (Fig. 2C and D). Notably, the addition of IL-15 did not significantly impact the development, CAR expression, or final purity of the engineered cells (Fig. 2C and D).

The differentiation of ^Allo/15^MCAR-NKT cells faithfully recapitulated the canonical developmental trajectory of human endogenous NKT cells [62, 63]. Throughout the six-week culture period, the cells consistently expressed pan-T cell markers, including CD5 and CD7, indicating robust T-lineage commitment (Figure S3A). As differentiation progressed, the population underwent a phenotypic shift from a naïve to a memory-like state, marked by upregulation of CD45RO and concomitant downregulation of CD45RA (Figure S3A). During the expansion phase, mature ^Allo/15^MCAR-NKT cells further acquired the NK-associated receptor CD161, reflecting the acquisition of a cytotoxic effector phenotype (Figure S3A).

Analysis of CD4 and CD8 co-receptor expression of ^Allo/15^MCAR-NKT cells revealed a developmental progression consistent with established NKT cell biology [62, 63]. The cells initially exhibited a double-negative (CD4⁻CD8⁻; DN) phenotype, transitioned through a double-positive (CD4⁺CD8⁺; DP) intermediate, and ultimately matured predominantly into DN or CD8 single-positive (CD8 SP) subsets (Figure S3B). The final ^Allo/15^MCAR-NKT product was largely enriched for DN and CD8 SP cells, with minimal CD4⁺ representation, consistent with prior in vitro NKT differentiation studies [40, 43, 44]. The preferential enrichment of DN and CD8 SP subsets, which are known for their potent cytotoxic capacity, is particularly advantageous for therapeutic applications in cancer immunotherapy [64–66].

In addition to high purity, the yield of allogeneic CAR-NKT cells was remarkable (Fig. 2E). Based on a linear calculation, a single CB unit containing 1–5 × 10^6^ CD34⁺ HSPCs yielded over 10^12 Allo/15^MCAR-NKT cells, which is sufficient for formulating approximately 1,000 to 10,000 therapeutic doses (Fig. 2E) [67–69]. This scalability supports the feasibility of treating multiple patients from a single manufacturing run, significantly lowering cost and advancing the potential for off-the-shelf application.

To confirm successful IL-15 incorporation and function, we compared cytokine secretion profiles between ^Allo^MCAR-NKT and ^Allo15^MCAR-NKT cells. ELISA revealed significantly higher secretion of human IL-15 from ^Allo15^MCAR-NKT cells (Fig. 2F). Moreover, these cells expressed elevated levels of IL-15–associated markers, including phosphorylated STAT5 (pSTAT5), Bcl-2, and Bcl-xL, indicating functional IL-15 signaling (Fig. 2G) [70, 71]. Given concerns about potential cytokine-driven autonomous proliferation, we evaluated the cytokine dependence of these cells using an in vitro dysregulated growth assay. Both ^Allo^MCAR-NKT and ^Allo15^MCAR-NKT cells failed to expand in the absence of exogenous IL-7 and IL-15, demonstrating that they retain cytokine dependence and suggesting a low risk of dysregulated or autonomous growth (Fig. 2H).

### Allogeneic MCAR-NKT cells exhibit enhanced cytotoxic and effector characteristics compared to conventional MCAR-T cells

To further characterize the phenotype and functionality of ^Allo/15^MCAR-NKT cells, we performed a side-by-side comparison with conventional MCAR-engineered T (MCAR-T) cells generated from healthy donor peripheral blood mononuclear cells (PBMCs) (Figures S3C-S3G). While MCAR-T cells exhibited high CAR expression levels (> 70%), a portion of the cells remained CAR-negative (Figures S3E and S3F). In contrast, nearly 100% of ^Allo/15^MCAR-NKT cells co-expressed the MCAR construct (Fig. 2B and C), thereby avoiding the heterogeneity commonly observed in conventional CAR-T products and ensuring a uniform, fully CAR-expressing therapeutic population.

We first examined the CD4/CD8 subpopulation profiles of ^Allo/15^MCAR-NKT cells in comparison to conventional MCAR-T cells. ^Allo/15^MCAR-NKT cells were predominantly composed of CD8 single-positive (SP) and double-negative (DN) populations, whereas conventional MCAR-T cells included both CD4 SP and CD8 SP subsets (Fig. 2I). Although CD4 SP NKT cells have been reported to play important roles in modulating immune responses and supporting antitumor immunity, such as through cytokine production and TME modulation, CD8 SP and DN NKT cells are more frequently associated with direct cytotoxicity and pro-inflammatory antitumor activity, making them well-suited for cancer immunotherapy [27, 37, 38, 42, 72]. Notably, the enrichment of CD8 SP and DN CAR-NKT cells appears to be an intrinsic feature of our ex vivo differentiation protocol, as similar phenotypes were observed in other allogeneic CAR-engineered NKT cell products generated using the same method (Fig. 2I) [39, 57]. Future modifications to the culture conditions may enable the generation of CD4 SP CAR-NKT cells, potentially broadening the therapeutic applications of this platform.

Flow cytometry analysis revealed that, compared to conventional MCAR-T cells, ^Allo/15^MCAR-NKT cells expressed significantly higher levels of natural killer receptors (NKRs), including NKG2D, DNAM-1, and NKp30 (Fig. 2J). This suggests that ^Allo/15^MCAR-NKT cells may possess intrinsic NKR-mediated cytotoxic potential against UEC tumor cells. Furthermore, ^Allo/15^MCAR-NKT cells produced elevated levels of proinflammatory cytokines (e.g., IFN-γ) and cytotoxic effector molecules (e.g., Perforin and Granzyme B), consistent with their predominant CD8 SP and DN phenotypes (Fig. 2I and J). These features collectively support their robust antitumor functionality.

We further characterized the transcriptional profile of ^Allo/15^MCAR-NKT cells using single-cell RNA sequencing (scRNA-seq) (Fig. 2K). Compared to conventional MCAR-T cells, ^Allo/15^MCAR-NKT cells exhibited reduced expression of T cell-associated genes, including *CD2*, *CD6*, *LAT*, *SIT1*, and *GRAP2* (Fig. 2K). Conversely, they expressed higher levels of NK cell-related genes such as *KLRC1*, *NCAM1*, *NCR3*, *KLRD1*, and *CD226* (Fig. 2K). Moreover, ^Allo/15^MCAR-NKT cells displayed elevated expression of effector and cytotoxicity-associated genes, including *CD69*, *HOPX*, *PRF1*, *GNLY*, *NKG7*, *NCR1*, and *GZMA* (Fig. 2K). Gene set enrichment analysis (GSEA) confirmed that, relative to MCAR-T cells, ^Allo15^MCAR-NKT cells exhibited significant enrichment of pathways linked to effector function, memory formation, and cytotoxic gene programs (Figure S3H). These transcriptional features suggest that ^Allo/15^MCAR-NKT cells possess potent NK-like effector and cytotoxic functions, which may contribute to their enhanced antitumor activity against UEC.

### Allogeneic MCAR-NKT cells simultaneously target UEC tumor cells and the tumor microenvironment (TME) in primary patient samples

We next assessed the antitumor efficacy of ^Allo/15^MCAR-NKT cells using primary UEC patient samples. The evaluation focused on two key aspects: (1) direct cytotoxicity against tumor cells, and (2) the ability to target the UEC TME, specifically through the elimination of immunosuppressive CD1d⁺ TAMs and MDSCs.

**Fig. 3:**
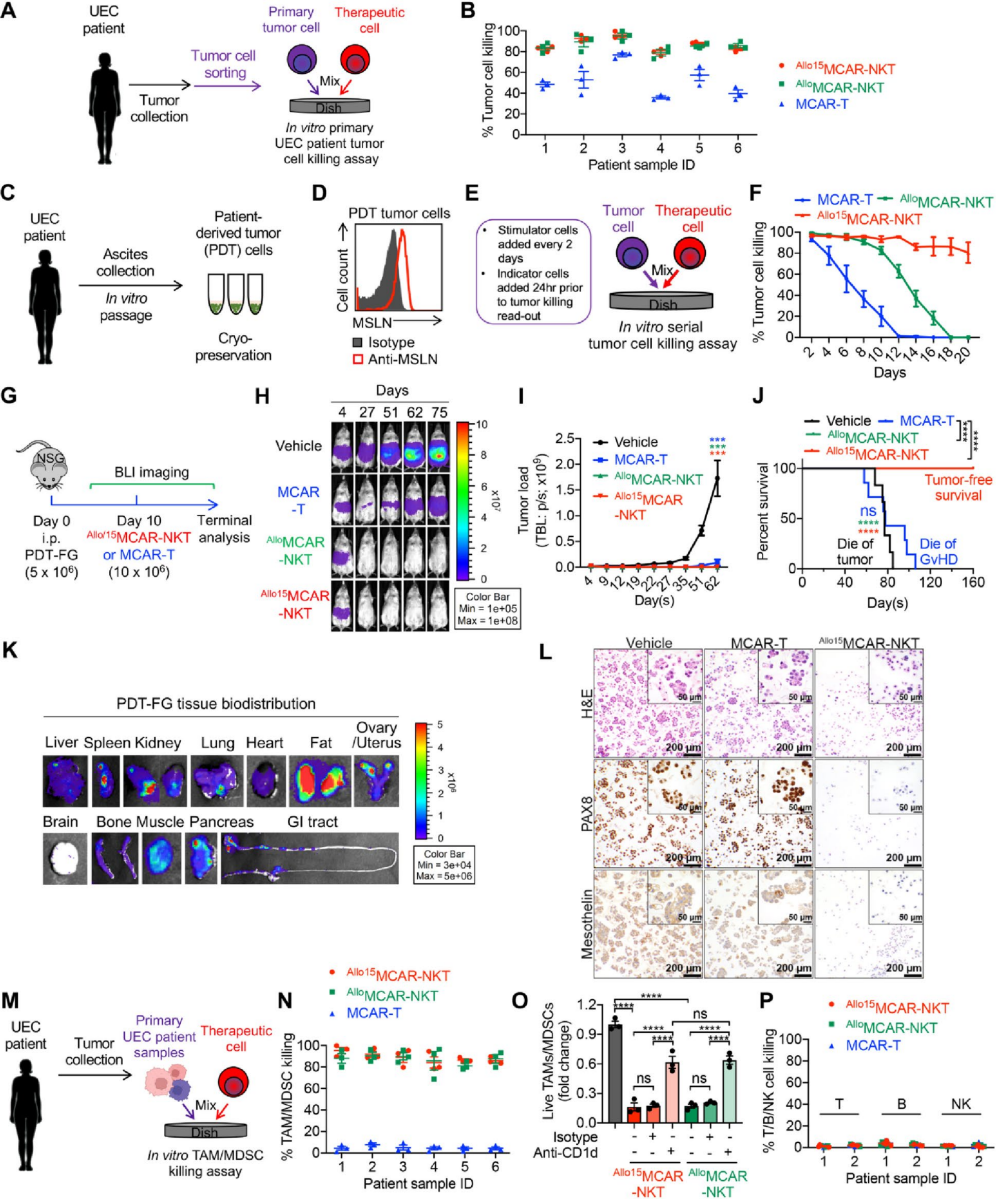
Allogeneic MCAR-NKT cells simultaneously target UEC tumor cells and the tumor microenvironment (TME) in primary cancer patient samples. **A** and **B** Studying the in vitro antitumor efficacy of ^Allo/15^MCAR-NKT cells using primary UEC patient samples. Conventional MCAR-T cells were included as a control. A total of 6 samples were included. **A** Experimental design. **B** Tumor cell killing data at 24 h (*n* = 3). **C**–**L** Studying the antitumor efficacy of ^Allo/15^MCAR-NKT cells using an UEC patient-derived tumor (PDT) cell line and its xenograft mouse model. Conventional MCAR-T cells were included as a control. **C** Diagram showing the generation of PDT cells. **D** FACS detection of MSLN expression on the PDT cells. (E and F) Studying the antitumor efficacy of ^Allo/15^MCAR-NKT cells using in vitro serial tumor cell killing assays. **E** Experimental design. **F** Tumor cell killing data (*n* = 4). **G**–**L** Studying the in vivo antitumor efficacy of ^Allo/15^MCAR-NKT cells in a PDT-FG xenograft model. **G** Experimental design. **H** BLI images showing tumor loads in experimental mice over time. **I** Quantification of H (*n* = 6-7). **J** Survival curve (*n* = 6-7). **K** BLI images showing the presence of PDT-FG tumor cells in various organs collected from the vehicle group of experimental mice on day 80. **L** H&E and immunohistochemistry (IHC) staining for PAX8 (a tumor lineage marker) and MSLN, showing the presence of PDT-FG tumor cells in the cell pellets generated from the intraperitoneal wash of experimental mice. **M**–**P** Studying ^Allo/15^MCAR-NKT cells targeting of UEC TME using primary UEC patient samples. Conventional MCAR-T cells were included as a control. A total of 6 primary UEC samples were analyzed. **M** Experimental design. **N** TAM/MDSC killing data at 24 h (*n* = 3). **O** TAM/MDSC killing data by ^Allo/15^MCAR-NKT cells with CD1d blockade at 24 h (*n* = 3). Data from 1 representative UEC sample are presented. **P** Killing data of the indicated UEC TME cell components at 24 h (*n* = 3). Data from 2 representative UEC samples are presented. Representative of 3 experiments. Data are presented as the mean ± SEM. ns, not significant, ***p < 0.001, ****p < 0.0001 by one-way ANOVA (**I** and **O**), or log rank (Mantel-Cox) test adjusted for multiple comparisons (**J**)

For the first set of assays, tumor cells were isolated from primary UEC patient samples and co-cultured with ^Allo/15^MCAR-NKT cells (Fig. 3A). Conventional MCAR-T cells served as controls. Across all six patient samples tested, ^Allo/15^MCAR-NKT cells demonstrated superior tumor cell killing compared to MCAR-T cells (Fig. 3B). This enhanced efficacy may be attributed to the high cytotoxic potential of ^Allo/15^MCAR-NKT cells, as well as their ability to recognize tumor targets via both CAR- and NKR-mediated mechanisms (Fig. 2J and K) [39, 57].

To further validate these findings, we established a stable MSLN⁺ patient-derived tumor (PDT) cell line from a primary UEC sample and engineered these cells to express firefly luciferase and enhanced green fluorescence protein (EGFP) dual reporters (FG) for real-time monitoring via flow cytometry and bioluminescence imaging (Fig. 3C and D). In an in vitro serial tumor cell killing assay, we compared the cytotoxic activity of ^Allo^MCAR-NKT, ^Allo15^MCAR-NKT, and conventional MCAR-T cells (Fig. 3E). All three cell products exhibited tumor-killing activity; however, MCAR-T cells were the least effective, while ^Allo15^MCAR-NKT cells demonstrated the most sustained tumor control (Fig. 3F). These results underscore the enhanced and prolonged antitumor activity of allogeneic MCAR-NKT cells conferred by IL-15 engineering.

To evaluate in vivo efficacy, we developed a UEC xenograft model using PDT-FG cells administered intraperitoneally (i.p.) to mimic peritoneal dissemination of UEC (Fig. 3G). Ten days post tumor inoculation, mice received i.p. injections of therapeutic cells, and tumor progression was monitored by bioluminescence imaging (BLI). Remarkably, both ^Allo^MCAR-NKT and ^Allo15^MCAR-NKT cells induced tumor eradication and prolonged survival (Fig. 3H and J). In contrast, while conventional MCAR-T cells achieved partial tumor suppression, eventual tumor relapse and mouse death were observed, likely due to both disease progression and xenogeneic graft-versus-host disease (GvHD), a known limitation of allogeneic CAR-T cell therapies in preclinical and clinical settings (Fig. 3H and J) [73–77].

Tissue-level BLI revealed widespread dissemination of PDT-FG tumor cells to multiple organs, including the liver, spleen, kidneys, lungs, ovaries, uterus, pancreas, and gastrointestinal tract (Fig. 3K). Notably, ^Allo/15^MCAR-NKT cells effectively eliminated tumor cells across all metastatic sites, demonstrating broad in vivo antitumor activity (Fig. 3H). H&E and IHC staining further confirmed the depletion of MSLN⁺PAX8⁺ tumor cells in the peritoneal cavity by ^Allo15^MCAR-NKT cells (Fig. 3L).

To investigate the ability of ^Allo/15^MCAR-NKT cells to modulate the TME, we co-cultured primary UEC patient-derived cells with each of the three therapeutic cells (Fig. 3M). While conventional MCAR-T cells failed to eliminate TAMs and MDSCs, both ^Allo^MCAR-NKT and ^Allo15^MCAR-NKT cells demonstrated efficient killing of these immunosuppressive myeloid cell populations (Fig. 3N). Notably, CD1d blockade markedly reduced the killing of TAMs and MDSCs by ^Allo^MCAR-NKT and ^Allo15^MCAR-NKT cells, demonstrating that this killing is largely dependent on CD1d/NKT TCR–mediated recognition (Fig. 3O). Importantly, ^Allo/15^MCAR-NKT cells did not target patient-derived T cells, B cells, or NK cells, consistent with their low or no expression of CD1d (Fig. 3P). These findings indicate that ^Allo/15^MCAR-NKT cells can selectively eliminate CD1d⁺ TAMs and MDSCs through NKT TCR-mediated recognition, without harming normal immune cells.

In conclusion, ^Allo/15^MCAR-NKT cells exhibit superior antitumor activity against UEC tumor cells and possess a unique ability to target key immunosuppressive components of the TME. Through the combined action of CAR-mediated, NKR-mediated, and TCR-mediated recognition, these cells effectively overcome tumor resistance and immune evasion. Together, these features position ^Allo/15^MCAR-NKT cells as a promising off-the-shelf cellular immunotherapy for UEC.

### Allogeneic MCAR-NKT cells demonstrate superior in vivo antitumor efficacy in human UEC xenograft mouse models

To further investigate the antitumor efficacy of ^Allo/15^MCAR-NKT cells, particularly under conditions of tumor antigen loss, a common mechanism of immune evasion following CAR-T cell therapy [78, 79], we employed two human UEC cell lines: HEC1B-FG (HEC1B cells engineered to express FG) and HEC1B-MSLN-FG (HEC1B-FG cells additionally overexpressing MSLN) (Figures S4A and S4B). These two cell lines allowed us to model and assess CAR antigen-dependent and antigen-independent tumor killing.

In in vitro cytotoxicity assays, conventional MCAR-T cells effectively eliminated MSLN⁺ tumor cells (HEC1B-MSLN-FG) but failed to kill MSLN⁻ tumor cells (HEC1B-FG), demonstrating exclusive dependence on CAR-mediated recognition (Figures S4C and S4D). In contrast, ^Allo15^MCAR-NKT cells effectively killed both tumor cell lines, with enhanced cytotoxicity against MSLN⁺ cells at lower effector-to-target (E: T) ratios, indicating the use of both CAR-dependent and NKR-mediated killing mechanisms (Figure S4D). This dual-targeting capability enables ^Allo15^MCAR-NKT cells to overcome antigen escape, a limitation for conventional CAR-T cells. Correlating with their superior cytotoxicity, ^Allo15^MCAR-NKT cells exhibited increased expression of activation markers such as CD25 and CD69, and elevated production of cytotoxic effectors including Perforin and Granzyme B (Figures S4E and S4F).

**Fig. 4:**
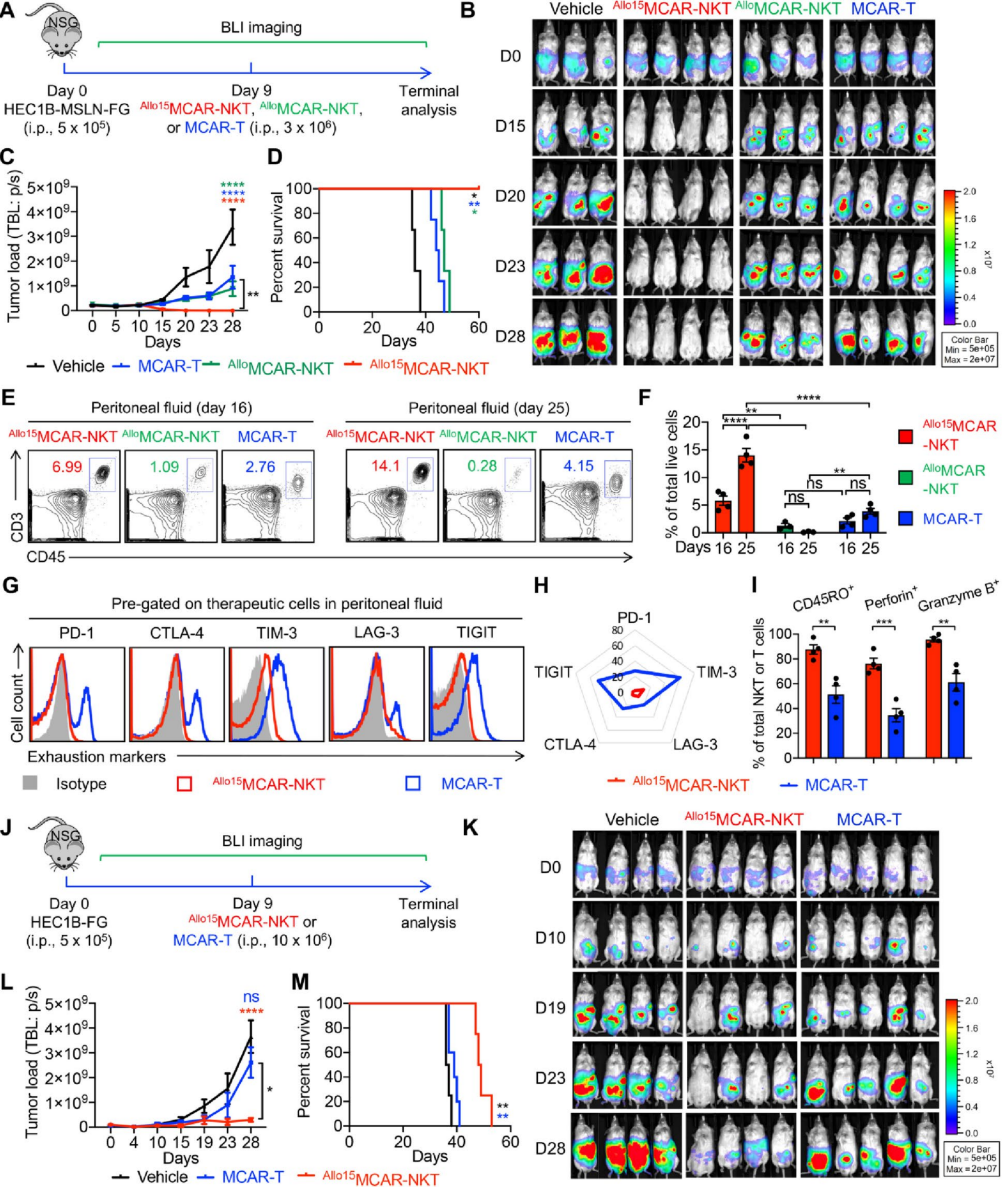
Allogeneic MCAR-NKT cells demonstrate superior in vivo antitumor efficacy in human UEC xenograft mouse models. **A**–**D** Studying the in vivo antitumor efficacy of ^Allo15^MCAR-NKT cells using a HEC1B-MSLN-FG human UEC xenograft NSG mouse model. Three types of therapeutic cells were included: ^Allo^MCAR-NKT, ^Allo15^MCAR-NKT, and conventional MCAR-T cells. 3 × 10^6^ therapeutic cells were administered intraperitoneally. **A** Experimental design. **B** BLI images showing the presence of tumor cells in experimental mice over time. **C** Quantification of (**B**) (*n* = 3–4). **D** Kaplan–Meier survival curves of experimental mice over time (*n* = 3–4). **E**–**I** Studying the in vivo persistence and phenotype of ^Allo15^MCAR-NKT cells. **E** FACS detection of the presence of therapeutic cells isolated from the peritoneal fluid of experimental mice at day 16 and 25. **F** Quantification of (**E**) (*n* = 3–4). **G** FACS detection of the exhaustion marker (i.e., PD-1, CTLA-4, TIM-3, LAG-3, and TIGIT) expression on ^Allo15^MCAR-NKT and MCAR-T cells isolated from the peritoneal fluid of experimental mice at day 25. **H** Quantification of (**G**) (*n* = 3–4). Radar plots are shown, representing the average values calculated from all mice. **I** FACS analyses of the T cell memory marker (i.e., CD45RO) expression and cytotoxic molecule (i.e., Perforin and Granzyme B) production in ^Allo15^MCAR-NKT and MCAR-T cells isolated from the peritoneal fluid of experimental mice at day 25 (*n* = 3–4). (J-M) Studying the in vivo antitumor efficacy of ^Allo15^MCAR-NKT cells using a HEC1B-MSLN-FG human UEC xenograft NSG mouse model. Two types of therapeutic cells were included: ^Allo15^MCAR-NKT and conventional MCAR-T cells. 10 × 10^6^ therapeutic cells were administered intraperitoneally. **J** Experimental design. **K** BLI images showing the presence of tumor cells in experimental mice over time. **L** Quantification of (K) (*n* = 4–5). **M** Kaplan–Meier survival curves of experimental mice over time (*n* = 4–5). Representative of 3 experiments. Data are presented as the mean ± SEM. ns, not significant, *p < 0.05, **p < 0.01, ***p < 0.001, ****p < 0.0001 by Student’s *t* test (**I**), by one-way ANOVA (**C**, **F**, and **L**) or by log rank (Mantel-Cox) test adjusted for multiple comparisons (**D** and **M**)

To validate these findings in vivo, we established two xenograft UEC mouse models using HEC1B-MSLN-FG or HEC1B-FG cells to represent CAR antigen–positive and –negative tumors, respectively. In the HEC1B-MSLN-FG model, low doses (3 × 10^6^) of therapeutic cells (^Allo^MCAR-NKT, ^Allo15^MCAR-NKT, and conventional MCAR-T) were administered intraperitoneally, and their antitumor activity was assessed (Fig. 4A). All three therapeutic products delayed tumor progression (Fig. 4B and D). However, only ^Allo15^MCAR-NKT cells achieved complete tumor eradication and long-term survival, whereas the other two therapies provided only partial tumor suppression and limited survival benefit (Fig. 4B and D).

Further analyses of therapeutic cells collected from the peritoneal cavity revealed distinct differences in in vivo dynamics. ^Allo15^MCAR-NKT cells exhibited robust expansion and long-term persistence, detectable until at least day 25, consistent with their superior antitumor effects (Fig. 4E and F). By contrast, ^Allo^MCAR-NKT cells failed to expand, likely due to the low dose of administration combined with the absence of IL-15 support, which may explain their limited efficacy (Fig. 4E and F). For conventional MCAR-T cells, tumor infiltration was observed; however, these cells displayed markedly reduced expansion compared to ^Allo15^MCAR-NKT cells, accompanied by elevated expression of exhaustion markers (PD-1, CTLA-4, TIM-3, LAG-3, and TIGIT), reduced levels of the T cell memory marker CD45RO, and significantly lower expression of cytotoxic molecules, including Perforin and Granzyme B (Fig. 4E and I). These features likely account for their inferior tumor control relative to ^Allo15^MCAR-NKT cells. Collectively, these results demonstrate that IL-15 engineering confers enhanced expansion, persistence, and effector function to allogeneic MCAR-NKT cells, establishing ^Allo15^MCAR-NKT cells as a lead therapeutic candidate for further development.

In the HEC1B-FG xenograft model, which mimics antigen escape, high doses (10 × 10^6^) of therapeutic cells, including ^Allo15^MCAR-NKT and conventional MCAR-T cells, were administered intraperitoneally to evaluate their antitumor activity (Fig. 4J). Conventional MCAR-T cells failed to control tumor growth or improve survival (Fig. 4K and M). In contrast, ^Allo15^MCAR-NKT cells significantly suppressed tumor progression and extended survival, likely through their intrinsic NKR-mediated CAR-independent cytotoxicity (Fig. 4K and M). These results highlight the ability of ^Allo15^MCAR-NKT cells to maintain antitumor activity even in the absence of CAR target antigens.

To further evaluate the superior antitumor potential of ^Allo15^MCAR-NKT cells, we assessed their activity against human ovarian cancer (OC) OVCAR8-FG cells both in vitro and in vivo. Although OC represents a distinct tumor type, it shares key biological features with UEC, including a common Müllerian origin, hormonal influences, and similarly immunosuppressive TME [5, 7]. Importantly, OVCAR8-FG cells expressed high levels of MSLN, making them susceptible to MCAR-based targeting (Figures S4G and S4H). Both MCAR-T and ^Allo15^MCAR-NKT cells effectively eliminated OVCAR8-FG cells; however, ^Allo15^MCAR-NKT cells demonstrated significantly enhanced cytotoxicity, likely due to their ability to engage multiple tumor-recognition mechanisms and their robust effector and cytolytic functions (Figure S4I). In an OVCAR8-FG xenograft mouse model, treatment with ^Allo15^MCAR-NKT cells resulted in superior tumor control and extended mouse survival compared with MCAR-T cells (Figures S4J-S4M). These findings suggest that ^Allo15^MCAR-NKT cells represent a versatile and potent therapeutic platform for the treatment of UEC, OC, and potentially other solid tumors.

### Allogeneic MCAR-NKT cells show high safety profile in human UEC xenograft mouse models

We then evaluated two critical safety aspects of ^Allo15^MCAR-NKT cells. The first was GvHD, a major concern in allogeneic cell therapies, as it can lead to severe immune-mediated damage to host tissues and multi-organ dysfunction [73, 76, 80]. The second was cytokine release syndrome (CRS), a common and potentially life-threatening adverse effect associated with CAR-T cell therapy, characterized by excessive systemic inflammation, high fever, hypotension, and elevated levels of proinflammatory cytokines such as IL-6 [81–84].

**Fig. 5:**
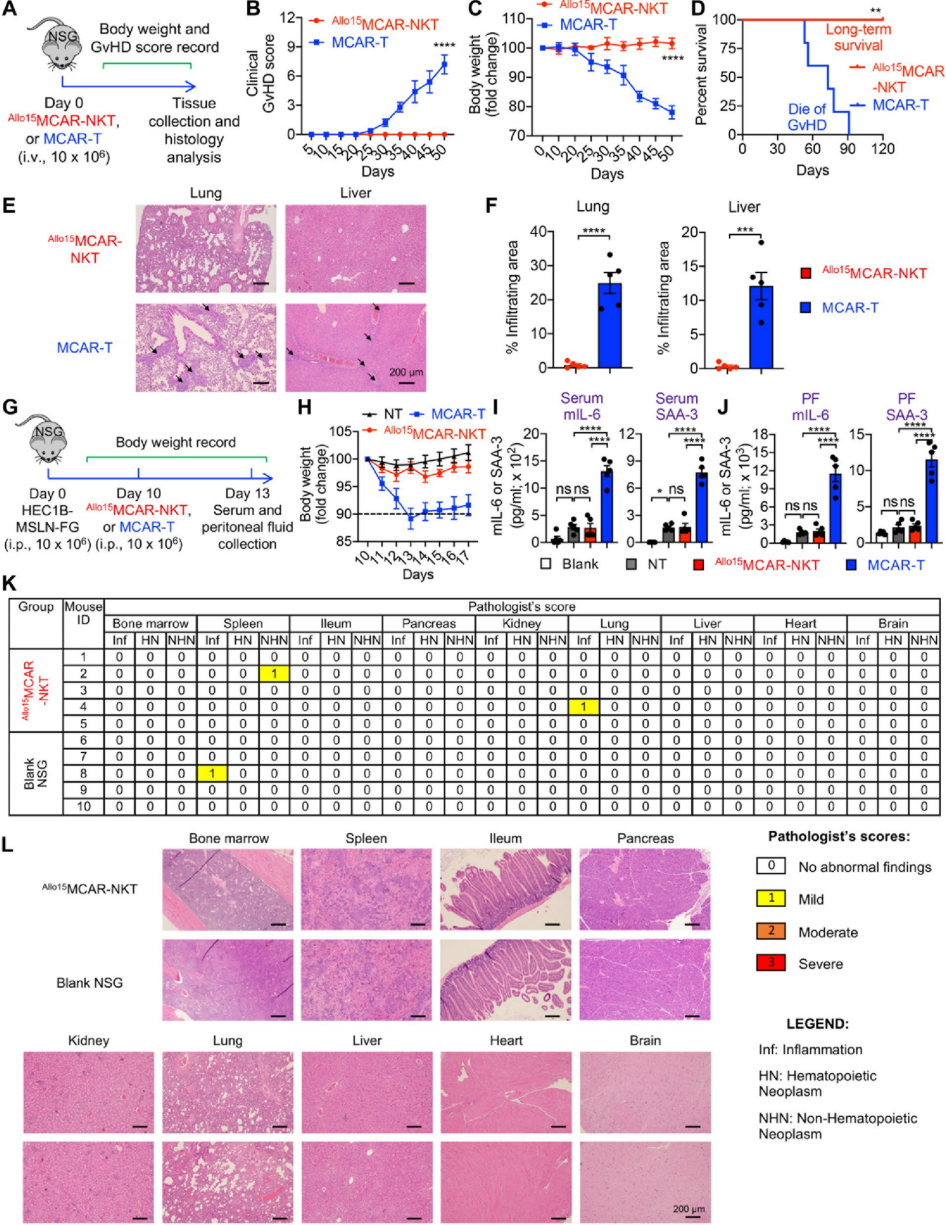
Allogeneic MCAR-NKT cells show high safety profile in human UEC xenograft mouse models. **A–F** Studying the GvHD risk of ^Allo15^MCAR-NKT cells using a human xenograft NSG mouse model. **A** Experimental design. **B** Clinical GvHD score recorded over time (*n* = 5). The score was calculated as the sum of individual scores of 6 categories (body weight, activity, posture, skin thickening, diarrhea, and dishevelment; score 0–2 for each category). **C** Body weight measured over time (*n* = 5). **D** Kaplan–Meier survival curves (*n* = 5). **E** H&E-stained tissue images. Tissue were collected from experimental mice on day 50. Scale bar, 200 μm. **F** Quantification of (**E**) (*n* = 5). **G**–**J** Studying the CRS response induced by ^Allo15^MCAR-NKT cells using a HEC1B-MSLN-FG human UEC xenograft NSG mouse model. **G** Experimental design. **H** Body weight of experimental mice over time (*n* = 5). **I** and **J** ELISA analyses of mouse IL-6 and SAA3 in mouse serum (**I**) or peritoneal fluid (**J**) (*n* = 5). Blank, samples collected from blank NSG mice; NT, samples collected from tumor-bearing mice receiving no therapeutic cell treatment; PF, peritoneal fluid. **K** and **L** Studying the long-term tissue toxicity of ^Allo15^MCAR-NKT cells. Tissues from experimental mice were collected 90 days after injection with 10 × 10^6 Allo15^MCAR-NKT cells. **K** Table showing the pathologist’s scores of individual mouse tissues. **L** H&E-stained tissue sections. Scale bar, 200 μm. Representative of 1 (**K** and **L**) or 3 (**A–J**) experiments. Data are presented as the mean ± SEM. ns, not significant, *p < 0.05, **p < 0.01, ***p < 0.001, ****p < 0.0001 by Student’s *t* test (**F**), one-way ANOVA (**I** and **J**), two-way ANOVA (**B** and **C**), or log rank (Mantel-Cox) test adjusted for multiple comparisons (**D**)

In contrast to conventional MCAR-T cells, ^Allo15^MCAR-NKT cells did not induce GvHD, as indicated by minimal clinical symptoms, reduced body weight loss, and prolonged survival (Fig. 5A and D). Histopathological analysis further revealed extensive immune cell infiltration in vital organs, including the lung and liver, in mice treated with conventional MCAR-T cells, whereas mice treated with ^Allo15^MCAR-NKT cells showed no significant infiltration (Fig. 5E and F). The absence of GvHD was also observed with HSPC-derived non–CAR-transduced NKT cells, indicating that this safety profile is an intrinsic property of the cell type (Figure S5). This lack of GvHD is likely attributable to the non-polymorphic nature of the invariant NKT TCR, which recognizes CD1d rather than mismatched MHC molecules, thereby avoiding alloreactivity [32–36, 85].

Furthermore, ^Allo15^MCAR-NKT cells exhibited a favorable cytokine release profile. Mice treated with ^Allo15^MCAR-NKT cells showed minimal CRS-associated toxicity, as evidenced by stable body weight and reduced levels of CRS biomarkers such as mouse IL-6 and serum amyloid A3 (SAA-3) (Fig. 5G and J). This reduced CRS risk may be due to the innate NK-like properties of ^Allo15^MCAR-NKT cells and their ability to target CRS-associated macrophages, as previously described (Fig. 2J and K, and Fig. 3M and O) [39, 86, 87].

Lastly, the enhanced in vivo persistence of ^Allo15^MCAR-NKT cells raises important translational considerations regarding long-term safety, particularly in tissues that express low levels of MSLN, such as pleura, pericardium, and peritoneum [88]. To address this, we evaluated potential long-term tissue toxicity of ^Allo15^MCAR-NKT cells. At 90 days post-infusion, multiple tissues were collected from experimental mice, and histopathological analysis revealed no evidence of toxicity across a range of vital organs, including bone marrow, spleen, lung, liver, brain, and others (Fig. 5K and L). These findings suggest that ^Allo15^MCAR-NKT cells maintain a favorable safety profile without causing on-target, off-tumor damage. Furthermore, as an allogeneic cell therapy, ^Allo15^MCAR-NKT cells are expected to be eliminated by host immune responses (e.g., T and NK cells) after the therapeutic window, similar to other allogeneic cell products [73, 74, 77, 89, 90]. This natural clearance further mitigates concerns regarding long-term toxicity.

In summary, ^Allo15^MCAR-NKT cells demonstrated robust antitumor efficacy against both CAR antigen–positive and antigen–negative UEC tumor cells, as well as an excellent safety profile, including minimal GvHD and CRS risk. These findings support the development of ^Allo15^MCAR-NKT cells as a potent and safe off-the-shelf cellular immunotherapy for UEC, with strong potential for clinical translation.

### Allogeneic MCAR-NKT cells exhibit enhanced antitumor capacity compared to MCAR-engineered NK cells

With the recent advancement of CAR-engineered natural killer (CAR-NK) cell therapy, particularly those derived from induced pluripotent stem cells (iPSCs), these allogeneic platforms have demonstrated encouraging efficacy and safety in both preclinical studies and early clinical trials [91–96]. To strengthen the translational relevance of our work, we conducted a direct comparison between CAR-NKT and CAR-NK cells. Specifically, we evaluated IL-15–enhanced ^Allo15^MCAR-NKT cells alongside human PBMC-derived IL-15–enhanced, MCAR-engineered NK (^PBMC15^MCAR-NK) cells.

For the generation of ^PBMC15^MCAR-NK cells, CD56⁺ NK cells were isolated from human PBMCs using MACS, stimulated with artificial APCs, and transduced with a lentiviral vector encoding both MCAR and human IL-15, achieving a transduction efficiency of ~ 30–40% (Figures S6A and S6B). Although CAR expression remained stable, ^PBMC15^MCAR-NK cells displayed limited expansion capacity, with only a 3–5-fold increase over one week of culture (Figure S6C). This restricted proliferative potential may hinder their feasibility as an off-the-shelf therapeutic product intended for broad patient application.

We next assessed the antitumor activity of the two engineered cell products using both short-term and long-term functional assays (Figures S6D-S6G). In the 24-hour cytotoxicity assay, both ^Allo15^MCAR-NKT and ^PBMC15^MCAR-NK cells exhibited potent tumor cell killing against MSLN⁺ HEC1B-MSLN-FG and MSLN⁻ HEC1B-FG human UEC cell lines (Figure S6E), indicating the capacity of both platforms to mediate tumor clearance through CAR-dependent and CAR-independent NKR-mediated mechanisms. However, in the serial tumor killing assay, ^Allo15^MCAR-NKT cells demonstrated superior durability, maintaining prolonged cytotoxic activity and achieving more sustained tumor control compared to ^PBMC15^MCAR-NK cells (Figure S6G).

In conclusion, ^Allo15^MCAR-NKT cells not only overcome the expansion limitations of PBMC-derived CAR-NK cells but also exhibit enhanced persistence and long-term tumor control, underscoring their potential as a more effective and scalable allogeneic cellular immunotherapy for UEC and other solid tumors.

## Discussion

In this study, we present a comprehensive preclinical evaluation of allogeneic MCAR-NKT cells, generated via a HSPC engineering platform combined with a six-week ex vivo differentiation culture (Fig. 2). This method yielded a highly pure (> 97%) population of functional CAR-NKT cells, characterized as NKT TCR⁺CD3⁺MCAR⁺ by flow cytometry (Fig. 2C and D). The high purity results from both the selective nature of the differentiation system, which exclusively supports NKT cell development, and the use of a single lentiviral construct encoding both the invariant NKT TCR and the MCAR, ensuring that all differentiated NKT cells uniformly express the CAR. Importantly, this manufacturing platform also supports high-yield cell production. Based on linear extrapolation, a single CB unit containing 1–5 × 10^6^ CD34⁺ HSPCs can generate over 10^12 Allo/15^MCAR-NKT cells, which corresponds to approximately 1,000 to 10,000 therapeutic doses based on current dosing strategies for CAR-T cell therapies [74, 97, 98]. This level of scalability supports the production of large batches for treating multiple patients, substantially lowering manufacturing costs and making true off-the-shelf application feasible.

Functionally, allogeneic MCAR-NKT cells exhibited potent antitumor activity against UEC tumor cells across multiple experimental platforms, including in vitro cytotoxicity assays and in vivo xenograft mouse models utilizing primary UEC patient samples, a patient-derived tumor cell line, and established UEC tumor cell lines (Figs. 3 and 4, and S4). Allogeneic MCAR-NKT cells consistently outperformed conventional MCAR-T cells in tumor killing, likely due to their enhanced effector function and dual tumor recognition mechanisms via both CAR and NKRs (Fig. 2J and K). This dual-targeting capability enabled effective elimination of tumor cells with low MSLN expression, addressing one of the key challenges of conventional CAR-T cell therapy—tumor antigen escape (Fig. 4J and M) [78, 79, 99]. Furthermore, IL-15-engineered ^Allo15^MCAR-NKT cells exhibited superior cytotoxicity and persistence compared to non-IL-15-engineered counterparts, underscoring the role of IL-15 in sustaining CAR-NKT functionality in vivo (Fig. 4A and I). Based on these findings, ^Allo15^MCAR-NKT cells were selected as the lead candidate for translational and clinical development.

In this study, we acknowledge the limitations of our in vivo xenograft mouse models, which utilized intraperitoneal tumor implantation and intraperitoneal delivery of therapeutic cells (Figs. 3G and 4A, and J). Our model is physiologically relevant as patients with UPSC in many cases can have disseminated intraperitoneal disease. In addition our model provides direct interaction between therapeutic and tumor cells, which we hope to recapitulate in clinical trials. We also recognize this model does not fully capture the complexities of solid tumor growth in all clinically relevant settings. To better evaluate translational potential, future investigations should employ subcutaneous or orthotopic tumor models. Such systems would provide other relevant disease sites to assess the capacity of ^Allo15^MCAR-NKT cells to traffic to tumor sites, infiltrate the TME, and maintain antitumor activity, which represent critical hurdles that remain major challenges for the clinical application of cell therapies in solid tumors including UEC [15, 17, 100, 101].

In addition to directly targeting tumor cells, allogeneic MCAR-NKT cells effectively modulated the immunosuppressive TME. Specifically, they selectively eliminated CD1d⁺ TAMs and MDSCs, without affecting CD1d⁻ immune cells such as T, B, and NK cells (Fig. 3M and O). This selective targeting preserves essential immune functions while overcoming immunosuppression, which remains a major barrier to successful immunotherapy in solid tumors [20, 53, 79, 102, 103].

Safety remains a central concern in CAR-based therapies. Allogeneic CAR-T cells require additional genetic modifications, such as TCR knockout, to prevent GvHD—modifications that may compromise cell fitness and persistence [74, 104–107]. In contrast, due to their invariant TCR that recognizes the non-polymorphic CD1d molecule, CAR-NKT cells are inherently GvHD-sparing, as demonstrated in preclinical xenogeneic models (Fig. 5A and F). This intrinsic safety feature eliminates the need for further genetic manipulation, enabling ready-to-use off-the-shelf therapeutic products. CRS is another major toxicity associated with conventional CAR-T cell therapy [81, 82]. Although CRS is less commonly reported in solid tumor trials, potentially due to limited efficacy and infiltration of CAR-T cells, our findings indicate that allogeneic MCAR-NKT cells pose a lower CRS risk than conventional CAR-T cells (Fig. 5G and J). This may be attributed to their innate NK-like phenotype and their capacity to eliminate CRS-promoting macrophages, which are known to amplify systemic inflammation via CD40–CD40L signaling interactions [39, 82, 86, 87, 108].

Although long-term toxicity studies indicate that ^Allo15^MCAR-NKT cells do not induce on-target, off-tumor effects (Fig. 5K and L), the potential for off-target toxicity related to the SS1 scFv used in the CAR remains an important consideration. A clinical trial led by the National Cancer Institute evaluated MCAR-T cells carrying the SS1 scFv in patients with mesothelin-expressing tumors, including malignant mesothelioma (NCT01583686). The therapy demonstrated a manageable safety profile, but clinical efficacy was limited, with only one of 15 patients achieving stable disease [109]. Another trial conducted in patients with malignant pleural mesothelioma and pancreatic ductal adenocarcinoma (NCT01355965) also reported a favorable safety profile, with transient tumor responses in 2 of 18 patients [110]. However, in a separate study, two cases of severe pulmonary toxicity were observed in the high-dose cohort of MCAR-T cells administered intravenously, highlighting that CAR-related toxicities remain a concern, particularly at higher doses intended to improve efficacy [111]. Importantly, our proposed ^Allo15^MCAR-NKT therapy belongs to the category of allogeneic cell products, which are ultimately eliminated by host immune responses, particularly host T and NK cells, after the therapeutic window. This natural clearance mechanism is expected to further mitigate the risk of long-term toxicity [76, 77, 104, 112].

In addition to conventional CAR-T cell therapy, CAR-NK cell therapy has emerged as a promising approach, offering notable advantages in terms of safety and versatility. Early clinical trial results have demonstrated encouraging efficacy of CAR-NK cells, particularly in B cell malignancies, where response rates are comparable to those achieved with CAR-T cells but with markedly reduced incidence of severe toxicities such as CRS, immune effector cell-associated neurotoxicity syndrome (ICANS), and GvHD [113–116]. Moreover, preclinical studies exploring diverse sources for generating CAR-NK cells, including PBMCs, umbilical cord blood, iPSCs, and NK cell lines such as NK-92, have further underscored the therapeutic potential of this platform [91, 93, 94, 117, 118]. NK-based treatments are being explored in therapy of uterine cancers, highlighting the potential of NK-based approaches for UEC [119]. In our study, we performed a direct comparison between allogeneic CAR-NKT cells and CAR-NK cells, both engineered with MCAR and IL-15 (Figure S6). Long-term serial tumor killing assays revealed that CAR-NKT cells exhibited superior persistence and sustained tumor suppression (Figure S6F and S6G), likely attributable to their T cell–like properties, as T cells are known to maintain longer in vivo persistence than NK cells [120–122]. Therefore, ^Allo15^MCAR-NKT cells may provide a more durable and clinically advantageous therapeutic strategy for UEC and potentially other solid tumors compared with PBMC-derived IL-15-enhanced CAR-NK cells.

Lastly, several important future directions emerge from this study. Here we utilize 6 clinical samples, 3 from ascites and 3 from dissociated uterine tumors. Future work can expand on testing additional clinical samples with a larger cohort from effusion, uterine curettage and hysterectomy specimens to provide a more comprehensive understanding of tumor heterogeneity and the immune landscape in UEC. These diverse sample sources would enable more robust validation of therapeutic targets and deepen insights into the interactions between tumor cells and the immune microenvironment. Second, the current preclinical evaluation primarily relied on NSG xenograft models, which, although useful, lack the complexity of a fully functional human immune system. Follow-up studies using humanized mouse models and, ideally, non-human primates will be essential to more accurately assess the persistence, immune interactions, and safety profile of allogeneic MCAR-NKT cells. Such efforts will be critical for translating these promising preclinical findings into clinically relevant and safe therapeutic strategies for patients. Finally, we acknowledge that the current cell culture platform is research-grade. Developing a GMP-compliant platform, including GMP facilities, GMP-grade lentiviral vectors, and scalable manufacturing systems such as G-REX, will be an essential next step to advance the clinical development of allogeneic MCAR-NKT cells.

## Conclusion

In conclusion, this study demonstrates the feasibility and therapeutic potential of allogeneic MCAR-NKT cells as a safe, scalable, and effective off-the-shelf immunotherapy for UEC. By simultaneously targeting tumor cells and the immunosuppressive microenvironment, while avoiding GvHD and tissue toxicity and minimizing CRS risk, allogeneic MCAR-NKT cells represent a transformative advance in the treatment of solid tumors. This work lays a strong foundation for future translational and clinical efforts aimed at bringing CAR-NKT cell therapy to patients with UEC and potentially other hard-to-treat malignancies.

## Supplementary Information

Supplementary Material

## Data Availability

The data generated and analyzed during this study are included in the article or can be accessed upon request from the corresponding authors. The scRNA-seq data generated in this study have been deposited in the public repository Gene Expression Omnibus Database (GSE270820 and GSE309020).

## Abbreviations

ADC: Antibody drug conjugate
^Allo^MCAR-NKT: Allogeneic mesothelin-targeting CAR-NKT
BLI: Bioluminescence live animal imaging
CAR: Chimeric antigen receptor
CAR-NK: CAR-engineered natural killer
CAR-NKT: CAR-engineered invariant natural killer T
CB: Cord blood
CRS: Cytokine release syndrome
DN: Double-negative
E:T ratio: Effector-to-target ratio
FAP: Fibroblast activation protein
GvHD: Graft-versus-host disease
H&E: Hematoxylin and eosin
HSPC: Hematopoietic stem and progenitor cell
i.p.: Intraperitoneally
IHC: Immunohistochemistry
NKR: Natural killer receptor
MCAR: MSLN-targeting CAR
MCAR-T: MCAR-engineered T
MDSC: Myeloid-derived suppressor cell
MSLN: Mesothelin
NK: Natural killer
NKT: Invariant natural killer T
PBMC: Peripheral blood mononuclear cell
pSTAT5: Phosphorylated STAT5
SAA-3: Serum amyloid A3
scRNA-seq: Single cell RNA sequencing
SP: Single-positive
TAM: Tumor-associated macrophage
TME: Tumor microenvironment
Treg: Regulatory T cell
UEC: Uterine endometrial carcinoma
UPSC: Uterine papillary serous carcinoma
