# Supplementary material for: Stem cell engineering for the generation of allogeneic CAR-directed natural killer T cells targeting endometrial carcinoma
Source: Exp Hematol Oncol. 2026 Jan 29;15:14. doi: 10.1186/s40164-026-00746-8 (PMC12857166; doi:10.1186/s40164-026-00746-8)
Supplement: Supplementary file 1 — Supplementary Material [file 40164_2026_746_MOESM1_ESM.pdf]

Supplementary Information

Stem Cell Engineering for the Generation of Allogeneic CAR-Directed Natural Killer T Cells Targeting Endometrial Carcinoma

Yan-Ruide Li[1,2,*], Gabriella A. DiBernardo[3,4,*], Yuning Chen[1,2], Xinyuan Shen[1,2], Ryan Hon[1,2], Lauryn E. Ruegg[3,4], Jie Huang[1,2], Adam Neal[3,4], Neda A. Moatamed[5], Sanaz Memarzadeh[3,4,6,7,8,9,§], Lili Yang[1,2,4,6,7,10,11,§]

Author Affiliation:

[1]Department of Microbiology, Immunology & Molecular Genetics, University of California, Los Angeles, Los Angeles, CA 90095, USA

[2]Department of Bioengineering, University of California, Los Angeles, Los Angeles, CA 90095, USA

[3]Department of Obstetrics and Gynecology, David Geffen School of Medicine, University of California, Los Angeles, Los Angeles, CA 90095, USA

[4]Eli and Edythe Broad Center of Regenerative Medicine and Stem Cell Research, University of California, Los Angeles, Los Angeles, CA 90095, USA

[5]Department of Pathology and Laboratory Medicine, David Geffen School of Medicine, University of California, Los Angeles, Los Angeles, CA 90095, USA

[6]Jonsson Comprehensive Cancer Center, David Geffen School of Medicine, University of California, Los Angeles, Los Angeles, CA 90095, USA

[7]Molecular Biology Institute, University of California, Los Angeles, Los Angeles, CA 90095, USA

[8]Department of Molecular and Medical Pharmacology, David Geffen School of Medicine, University of California, Los Angeles, Los Angeles, CA 90095, USA

[9]The VA Greater Los Angeles Healthcare System, Los Angeles, CA 90073, USA

[10]Parker Institute for Cancer Immunotherapy, University of California, Los Angeles, Los Angeles, CA 90095, USA

[11]Goodman-Luskin Microbiome Center, University of California, Los Angeles, Los Angeles, CA 90095, USA

[*] These authors contributed equally

[§]Corresponding authors. Email: liliyang@ucla.edu (L.Y.), smemarzadeh@mednet.ucla.edu (S.M.)

A

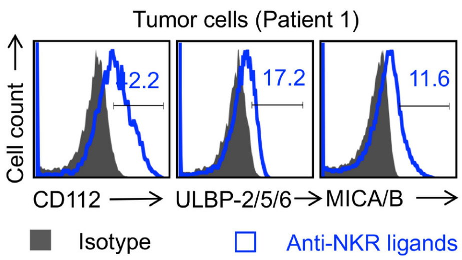

B

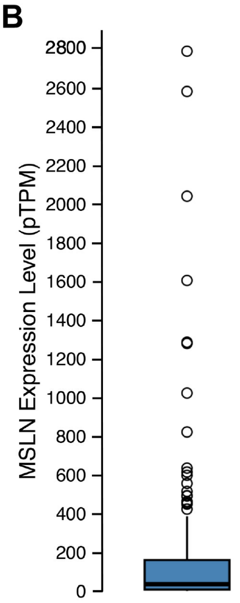

MSLN expression on tumor cells from patients with uterine endometrial carcinoma		
MSLN expression level	Expression value	Patient number
High	> 1,000 TPM	7
Medium	10-1,000 TPM	114
Low	0.5-10 TPM	44
Below cutoff	< 0.5 TPM	10

Figure S1. Profiling primary UEC patient samples; related to Figure 1.

(A) FACS plots showing the NKR ligand expression on primary UEC tumor cells.

(B) MSLN mRNA expression across 175 UEC patient samples from the Human Protein Atlas (https://www.proteinatlas.org/ENSG00000102854-MSLN/cancer/endometrial+cancer). The box shows the median and interquartile range (IQR), whiskers extend to 1.5×IQR, and circles denote outliers. pTPM, protein-coding transcripts per million.

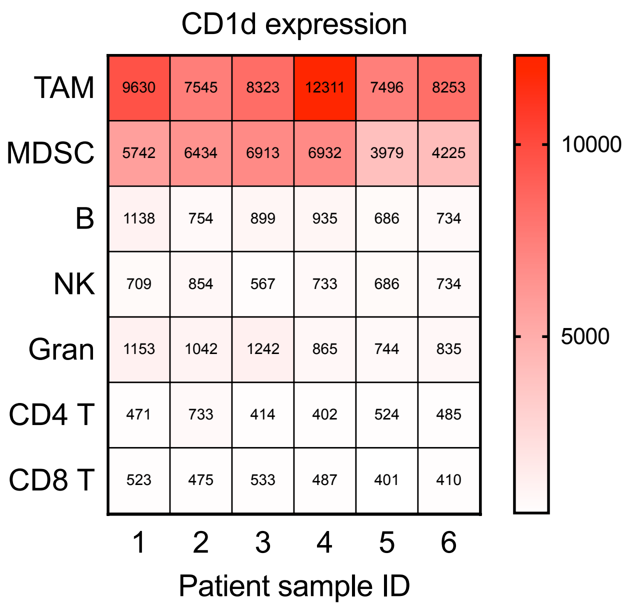

Figure S2. Heatmap showing the CD1d expression on the indicated immune cells; related to Figure 1.

The numbers represent the mean fluorescence intensity (MFI) of CD1d expression.

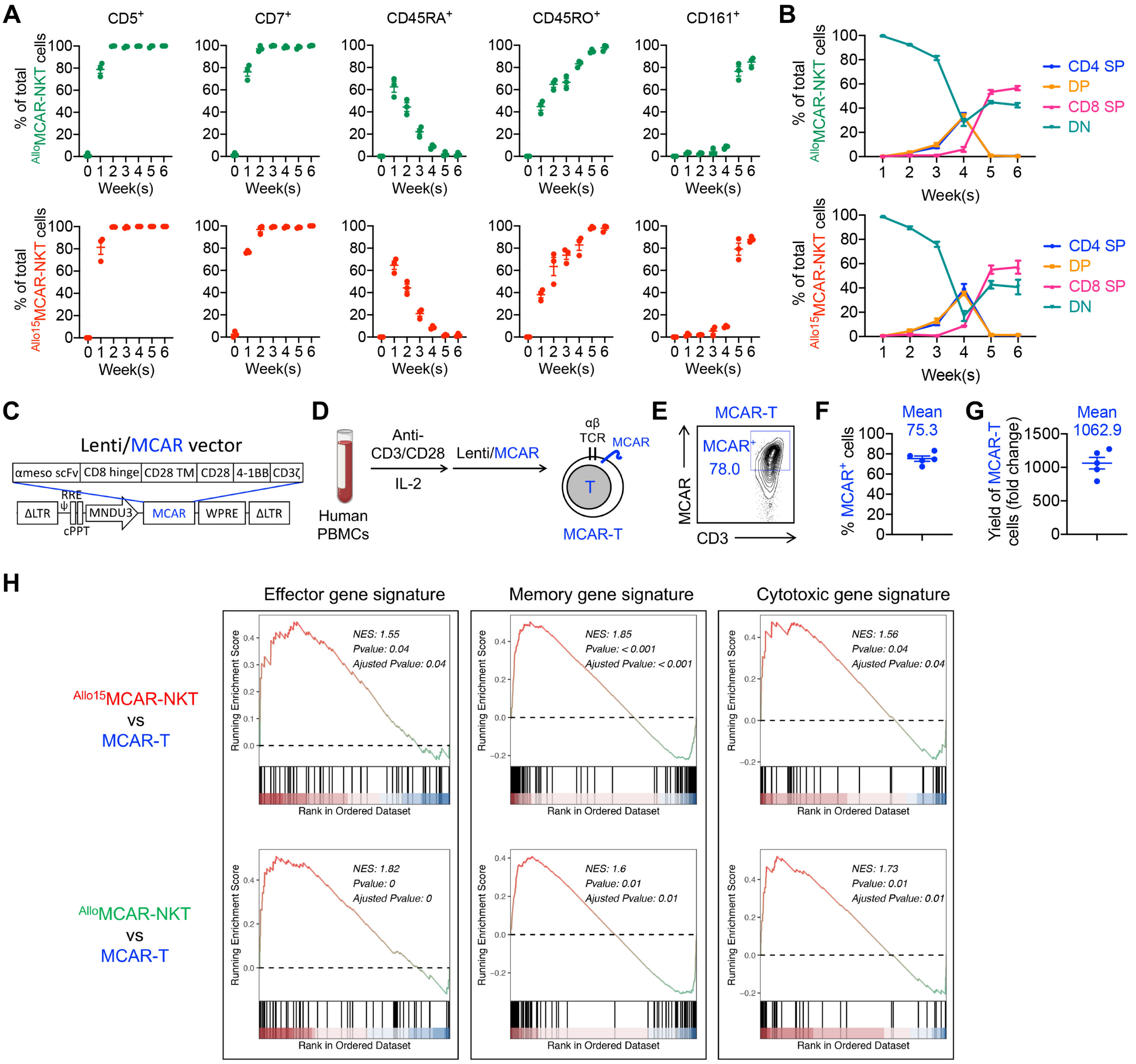

Figure S3. Generation and characterization of $^{Allo/15}$MCAR-NKT cells; related to Figure 2.

(A-B) Monitoring the differentiation of $^{Allo/15}$MCAR-NKT cells over the six-week culture period. (A) Percentage of $^{Allo/15}$MCAR-NKT cells expressing the indicated markers (i.e., CD5, CD7, CD45RA, CD45RO, and CD161) among total $^{Allo/15}$MCAR-NKT cells during the 6-week culture (n = 3; n indicates different CB donors). (B) Percentage of the subpopulations of $^{Allo/15}$MCAR-NKT cells during the 6-week culture (n = 3; n indicates different CB donors).

(C-G) Generating PBMC-derived conventional MCAR-T cells. (C) Schematics showing the design of Lenti/MCAR vector. (D) Schematics showing the generation of conventional MCAR-T cells from healthy donor PBMCs. (E) FACS plot showing the MCAR expression on MCAR-T cells. (F) Quantification of CAR^{+} proportion of MCAR-T cells (n = 5). (G) Yield of MCAR-T cells (n = 5).

(H) GSEA plots showing the enrichment of gene signatures in $^{Allo/15}$MCAR-NKT cells relative to MCAR-T cells, highlighting pathways associated with effector function, memory, and cytotoxicity.

Representative of 1 (H) and over 5 (A-G) experiments. Data are presented as the mean ± SEM.

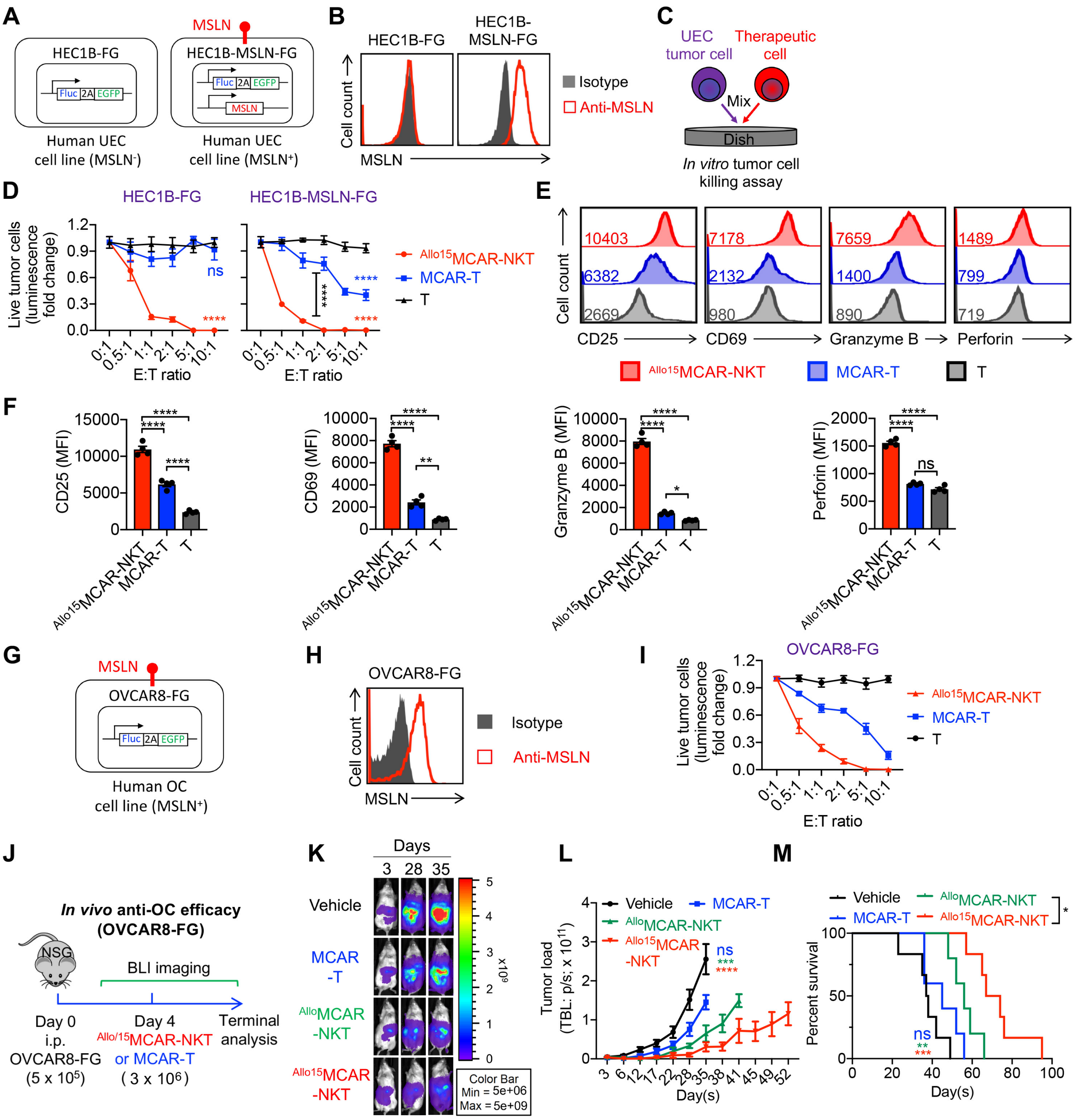

Figure S4. Tumor targeting efficacy and mechanisms of ^{Allo15}MCAR-NKT cells; related to Figure 4.

(A-F) *In vitro* UEC tumor targeting by ^{Allo15}MCAR-NKT cells. (A) Schematics showing the indicated human UEC cell lines. HEC1B-FG, HEC1B cell line engineered to overexpress the firefly luciferase and green fluorescence protein dual reporters (FG); HEC1B-MSLN-FG, HEC1B-FG cell line engineered to overexpress human MSLN. (B) FACS detection of MSLN expression on the indicated UEC cells. (C) Schematics showing the *in vitro* tumor cell killing assay. (D) Tumor cell killing data at 24 h (n = 4). (E) FACS detection of surface activation

markers (i.e., CD25 and CD69) as well as intracellular cytotoxic molecules (i.e., Perforin and Granzyme B) in the indicated therapeutic cells. (F) Quantification of (E) (n = 4).

(G-I) *In vitro* ovarian cancer (OC) targeting by ^{Allo15}MCAR-NKT cells. (G) Schematics showing the OVCAR8-FG human OC cell line. (H) FACS detection of MSLN expression on OVCAR8-FG tumor cells. (I) Tumor cell killing data at 24 h (n = 4).

(J-M) *In vivo* OC targeting by ^{Allo15}MCAR-NKT cells. (J) Experimental design. (K) BLI images showing the presence of tumor cells in experimental mice over time. (L) Quantification of (K) (n = 5-6). (M) Kaplan-Meier survival curves of experimental mice over time (n = 5-6).

Representative of 3 experiments. Data are presented as the mean ± SEM. ns, not significant, *p < 0.05, **p < 0.01, ****p < 0.0001 by by one-way ANOVA (F and L), two-way ANOVA (D), or log rank (Mantel-Cox) test adjusted for multiple comparisons (M).

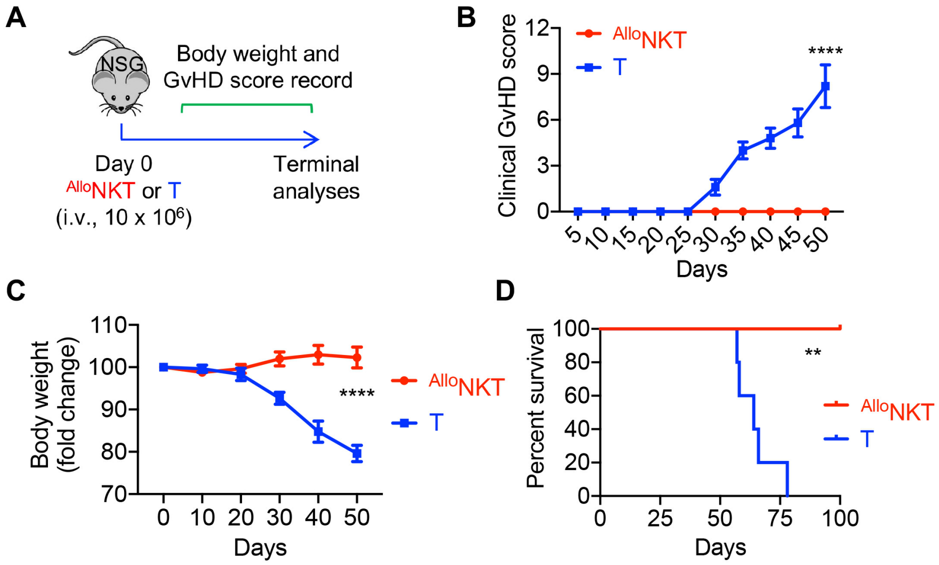

Figure S5. Studying the GvHD risk of allogeneic HSPC-derived NKT (^{Allo}NKT) cells using a human xenograft NSG mouse model; related to Figure 5.

(A) Experimental design.

(B) Clinical GvHD score recorded over time (n = 5). The score was calculated as the sum of individual scores of 6 categories (body weight, activity, posture, skin thickening, diarrhea, and dishevelment; score 0-2 for each category).

(C) Body weight measured over time (n = 5).

(D) Kaplan–Meier survival curves (n = 5).

Representative of 2 experiments. Data are presented as the mean ± SEM. $^{**}p < 0.01$, $^{****}p < 0.0001$ by two-way ANOVA (B and C; statistics shown for day 50) or by log rank (Mantel-Cox) test adjusted for multiple comparisons (D).

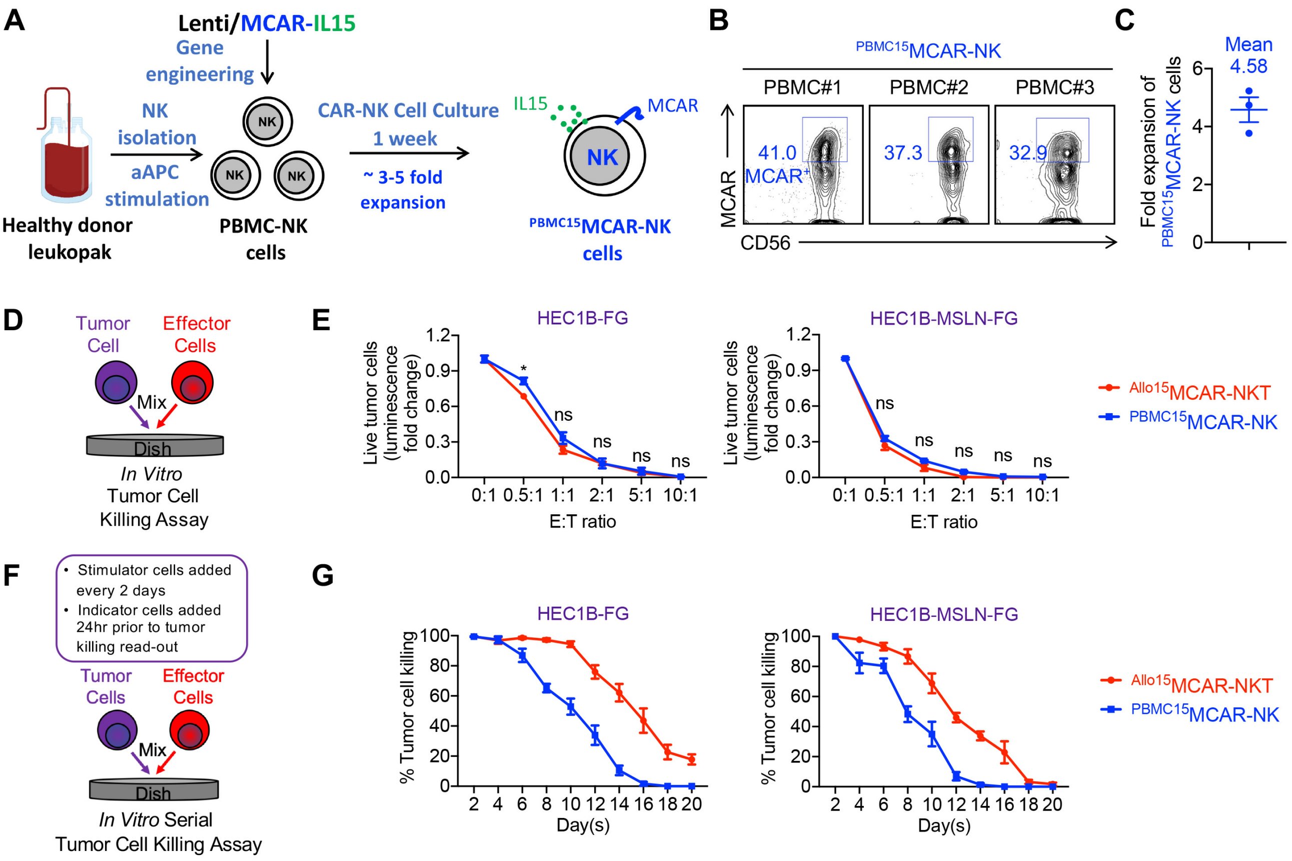

Figure S6. Comparison between $^{\text{Allo15}}$MCAR-NKT and human PBMC-derived IL-15-enhanced MCAR-engineered NK ($^{\text{PBMC15}}$MCAR-NK) cells.

(A) Schematics showing the generation of $^{\text{PBMC15}}$MCAR-NK cells from healthy donor PBMCs.

(B) FACS plots showing the MCAR expression on $^{\text{PBMC15}}$MCAR-NK cells. Data from three independent PBMC donors are presented.

(C) Fold expansion of $^{\text{PBMC15}}$MCAR-NK cells (n = 3).

(D and E) Comparison of the *in vitro* antitumor efficacy of $^{\text{Allo15}}$MCAR-NKT and $^{\text{PBMC15}}$MCAR-NK cells using a 24-hour *in vitro* tumor cell killing assay. (D) Experimental design. (E) Tumor cell killing data at 24 h (n = 4).

(F and G) Comparison of the *in vitro* antitumor efficacy of $^{\text{Allo15}}$MCAR-NKT and $^{\text{PBMC15}}$MCAR-NK cells using a long-term *in vitro* serial tumor cell killing assay. (E) Experimental design. (F) Tumor cell killing data (n = 4).

Representative of 3 experiments. Data are presented as the mean ± SEM. ns, not significant; $^{*}p < 0.05$, by two-way ANOVA (E).

Table S1. Primary UEC patient information

Patient	Age range	Sample type	Diagnosis	Treatment status
1	60-64	Ascites	Uterine high-grade serous carcinoma (HGSC)	Chemonaive
2*	65-69	Ascites	Uterine HGSC	Chemonaive
3	55-59	Dissociated tumor uterus origin	Ovarian + uterine HGSC	Chemonaive
4	70-74	Dissociated tumor uterus origin	Uterine HGSC	Chemonaive
5	65-69	Dissociated tumor uterus origin	Uterine endometroid + HGSC	Chemonaive
6	65-69	Ascites	Uterine HGSC	Recurrent/Persistent

*Sample used to generate PDT cell line.

Additional methods

Mice

NOD.Cg-Prkdc^{SCID} Il2rg^{tm1Wjl} /SzJ (NOD/SCID/IL-2Rγ$^{-/-}$, NSG) mice were purchased from The Jackson Laboratory (Strain #:005557; RRID:IMSR_JAX:005557), and maintained in the animal facilities of UCLA under the following housing conditions: temperature ranging from 20 °C to 26 °C, humidity maintained at 30% to 70%, a light cycle of On at 6:00 am and Off at 6:00 pm, and room pressure set to negative. 6-10 weeks old mice were used for all experiments unless otherwise indicated. Notably, no differences were observed in tumor growth, survival, or disease symptoms between male and female mice in the xenograft models used in this study. All animal experiments were approved by the Institutional Animal Care and Use Committee of UCLA. All mice were bred and maintained under specific pathogen-free conditions, and all experiments were conducted in accordance with the animal care and use regulations of the Division of Laboratory Animal Medicine at the UCLA. Experimental mice were randomly assigned to treatment groups to avoid statistically significant differences in the baseline tumor burden.

Media and reagents

The X-VIVO 15 Serum-Free Hematopoietic Cell Medium (cat. no. 04418Q) was purchased from Lonza. The StemSpan™ T Cell Generation Kit (cat. no. 09940), comprising the StemSpan™ SFEM II Medium (cat. no. 09605), the StemSpan™ Lymphoid Progenitor Expansion Supplement (cat. no. 09915), the StemSpan™ LPMS (cat. no. 09930), the StemSpan™ Lymphoid Progenitor Differentiation Coating Material (cat. no. 09925), and the ImmunoCult™ Human CD3/CD28/CD2 T Cell Activator (cat. no.10970), and MethoCult™ H4330 MethycelluloseBased Medium (cat. no. 04330) were purchased from StemCell Technologies. The CTS™ OpTmizer™ T-Cell Expansion SFM (no phenol red, bottle format, cat. no. A3705001), the RPMI 1640 cell culture medium (cat. no. MT10040CV), and the DMEM cell culture medium (cat. no. MT10013CV) were purchased from Thermo Fisher Scientific. The CryoStor® Cell Cryopreservation Media CS10 (cat. no. C2874) and Iscove's Modified

Dulbecco's Medium (cat. no. I3390) was purchased from MilliporeSigma. The C10 medium was made of RPMI 1640 cell culture medium, supplemented with FBS (10% vol/vol), P/S/G (1% vol/vol), MEM NEAA (1% vol/vol), HEPES (10 mM), Sodium Pyruvate (1 mM), Beta-Mercaptoethanol (β-ME) (50 μM), and Normocin (100 μg/ml). The homemade D10 medium was made of DMEM supplemented with FBS (10% vol/vol), P/S/G (1% vol/vol), and Normocin (100 μg/ml). The homemade R10 medium was made of RPMI supplemented with FBS (10% vol/vol), P/S/G (1% vol/vol), and Normocin (100 μg/ml).

α-Galactosyl ceramide (αGC, KRN7000, cat. no. 867000) was purchased from Avanti Polar Lipids. Recombinant human IL-2 (cat. no. 200-02), IL-3 (cat. no. 200-03), IL-7 (cat. no. 200-07), IL-15 (cat. no. 200-15), IFN-γ (cat. no. 300-02), Flt3 ligand (Flt3L, cat. no. 300-19), macrophage colony stimulating factor (M-CSF, cat. no. 300-25), stem cell factor (SCF, cat. no. 300-07), and thrombopoietin (TPO, cat. no. 300-18) were purchased from Peprotech. Fetal Bovine Serum (FBS, lot no. 2087050) were purchased from Gibco and β-ME (cat. no. 1610710) were purchased from Bio-Rad. Penicillin Streptomycin-Glutamine (P/S/G, cat. no. 10-378-016), MEM nonessential amino acids (NEAA, cat. no. 11-140-050), HEPES Buffer Solution (cat. no. 15630080), and Sodium Pyruvate (cat. no. 11360070) were purchased from Gibco. Normocin was purchased from InvivoGen (cat. no. NC9390718).

Antibodies and flow cytometry

Fluorochrome-conjugated antibodies specific for human CD1d (Clone 51.1, PE-Cy7 or APC-conjugated, 1:50, cat. no. 350310 or 350308), CD3 (Clone HIT3a, Pacific Blue, PE, or PE-Cy7-conjugated, 1:500, cat. no. 300330, 300308, or 300316), CD4 (Clone OKT4, PE-Cy7, PerCP or FITC-conjugated, 1:500, cat. no. 317414, 317432 or 317408), CD5 (Clone UCHT2, PerCP-conjugated, 1:200, cat. no 300618), CD7 (Clone CD7-6B7, APC-conjugated, 1:200, cat no. 343108), CD8 (Clone SK1, PE, APC-Cy7, or APC-conjugated, 1:300, cat. no. 344706, 344714 or 344722), CD14 (Clone HCD14, Pacific Blue-conjugated, 1:100, cat. no. 367122), CD19 (Clone HIB19, APC-Cy7-conjugated, 1:200, cat. no. 302218), CD25 (Clone BC96, PE-conjugated, 1:100, cat. no. 302606), CD34 (Clone 581, PerCP-conjugated, 1:500, cat. no.

343520), CD31 (Clone WM59, FITC-conjugated, 1:100, cat. no. 989002), CD45 (Clone HI30, PerCP, FITC, or Pacific Blue-conjugated, 1:500, cat. no. 982318, 982316, or 982306), CD69 (Clone FN50, PE-Cy7 or PerCP-conjugated, 1:50, cat. no. 310912 or 310928), CD112 (Clone TX31, PE-conjugated, 1:250, cat. no. 337410), CD155 (Clone SKII.4, PE-Cy7-conjugated, 1:250, cat. no. 337614), CD11b (Clone ICRF44, FITC-conjugated, 1:500, cat. no. 982614), MICA/MICB (Clone 6D4, PE or APC-conjugated, 1:25, cat. no. 320906 or 320908), CD161 (Clone W18070C, APC-conjugated, 1:50, cat. no. 307512), CD45RO (Clone UCHL1, FITC-conjugated, 1:200, cat. no. 983110), CD45RA (Clone HI100, PE-Cy7-conjugated, 1:500, cat. no. 983006), PD-1 (Clone A17188A, PE or FITC-conjugated, 1:25, cat. no. 379210 or 379206), TIM-3 (Clone A18087E, APC-conjugated, 1:25, cat. no. 364804), LAG-3 (Clone 7H2C65, PE-Cy7-conjugated, 1:25, cat. no. 369208), CTLA-4 (Clone L3D10, PE-Cy7-conjugated, 1:50, cat. no. 349914), TIGIT (Clone A15153G, APC-Cy7-conjugated, 1:50, cat. no. 372734), NKG2D (Clone 1D11, PE-Cy7-conjugated, 1:50, cat. no. 320812), DNAM-1 (Clone 11A8, APC-conjugated, 1:50, cat. no. 338312), NKp30 (Clone P30-15, APC-conjugated, 1:50, cat. no. 325210), IFN-γ (Clone B27, PE-Cy7-conjugated, 1:50, cat. no. 506518), Granzyme B (Clone QA16A02, APC-conjugated, 1:2000 or 1:5000, cat. no. 372204), and Perforin (Clone dG9, PE-Cy7-conjugated, 1:50 or 1:100, cat. no. 308126), were purchased from BioLegend. Fluorochrome-conjugated antibodies specific for human iNKT TCR Vα24-Jβ18 (Clone 6B11, PE-conjugated, 1:20, cat. no. 552825) were purchased from BD Biosciences. Fluorochrome-conjugated antibodies specific for human fibroblast activation protein FAP (Clone 427819, PE-conjugated, 1:100, cat. no. FAB3715P), ULBP-1 (Clone 170818, PE-conjugated or unconjugated, 1:25, cat. no. FAB1380P or MAB1380), ULBP-2,5,6 (Clone 165903, APC-conjugated, 1:25, cat. no. FAB1298A), and MSLN (Clone 420411, APC-conjugated, 1:20, cat. no. FAB32652A), were purchased from R&D Systems. A goat anti-mouse IgG F(ab')2 secondary antibody (cat. no. A-11001) was purchased from ThermoFisher. Fixable Viability Dye eFluor506 (e506, 1:500, cat. no. 65-0866-14) was purchased from Affymetrix eBioscience; mouse Fc Block (anti-mouse CD16/32, cat. no. 553141) was purchased from BD Biosciences; and human Fc Receptor Blocking Solution (TrueStain FcX) was purchased from BioLegend

(cat. no. 422302). In our study, note the use of antibodies with identical clones but differing conjugated fluorochromes, with one typical antibody listed herein.

All FACS staining was performed following manufacturers' provided protocols. Appropriate isotype staining controls were used for all staining procedures. Stained cells were analyzed using a MACSQuant Analyzer 10 flow cytometer (Miltenyi Biotech), following the manufacturer's instructions. FlowJo software version 9 (BD Biosciences) was used for data analysis.
